# Mitochondrial general control of amino acid synthesis 5 like 1 promotes nonalcoholic steatohepatitis development through ferroptosis‐induced formation of neutrophil extracellular traps

**DOI:** 10.1002/ctm2.1325

**Published:** 2023-07-06

**Authors:** Tingting Lv, Xiaofeng Xiong, Wei Yan, Mei Liu, Hongwei Xu, Qin He

**Affiliations:** ^1^ Department of Gastroenterology Shandong Provincial Hospital Affiliated to Shandong First Medical University Jinan China; ^2^ Department of Cancer Center Shandong Provincial Hospital Affiliated to Shandong First Medical University Jinan China; ^3^ Department of Gastroenterology Institute of Liver and Gastrointestinal Diseases, Tongji Hospital of Tongji Medical College, Huazhong University of Science and Technology Wuhan China

**Keywords:** ferroptosis, GCN5L1, mitochondrial permeability transition pores, neutrophil extracellular traps, nonalcoholic steatohepatitis

## Abstract

**Background:**

Mitochondria play central roles in metabolic diseases including nonalcoholic steatohepatitis (NASH). However, how mitochondria regulate NASH progression remains largely unknown. Our previous findings demonstrate that mitochondrial general control of amino acid synthesis 5 like 1 (GCN5L1) is associated with mitochondrial metabolism. Nevertheless, the roles of GCN5L1 in NASH are unclear.

**Aims and methods:**

The GCN5L1 expression was detected in the fatty livers of NASH patients and animals. Hepatocyte‐specific GCN5L1 deficiency or overexpression mice were used to induce NASH models by feeding with a high‐fat/high‐cholesterol or methionine‐choline deficient diet. The molecular mechanisms underlying GCN5L1‐regulated NASH were further explored and verified in mice.

**Results and conclusions:**

GCN5L1 expression was increased in NASH patients. Upregulated GCN5L1 level was also illustrated in NASH mice. Mice with hepatocyte‐specific GCN5L1 conditional knockout improved the inflammatory response compared to GCN5L1^flox/flox^ mice. However, overexpression of mitochondrial GCN5L1 augmented the inflammatory response. Mechanically, GCN5L1 acetylated CypD and enhanced its binding with ATP5B, which induced the opening of mitochondrial permeability transition pores and the release of mitochondrial ROS into the cytoplasm. The increased ROS promoted ferroptosis of hepatocytes and induced accumulation of high mobility group box 1 in the microenvironment, which recruited neutrophils and induced the generation of neutrophil extracellular traps (NETs). NETs block impaired GCN5L1‐induced NASH progression. Furthermore, the upregulation of GCN5L1 in NASH was contributed by lipid overload‐induced endoplasmic reticulum stress. Together, mitochondrial GCN5L1 has a vital function in promoting NASH progression by regulating oxidative metabolism and the hepatic inflammatory microenvironment. Thus, GCN5L1 might be a potential intervention target in NASH treatment.

## Graphical abstract

1. General control of amino acid synthesis 5 like 1 (GCN5L1) expression is increased in nonalcoholic steatohepatitis (NASH) patients and positively relates with the severity of NASH.
2. GCN5L1 promotes ferroptosis of hepatocytes by acetylating CypD and releases mitochondrial ROS by opening mitochondrial permeability transition pores.
3. High mobility group box 1 recruits neutrophils and induces the generation of NETs, which promotes NASH progression.
4. Lipid‐induced endoplasmic reticulum stress contributes to the upregulation of GCN5L1 by ATF4.

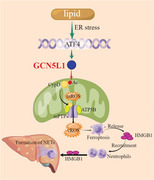

## 1 INTRODUCTION

As an immunometabolic disease, nonalcoholic fatty liver disease (NAFLD) is considered a public health challenge because of the changes in dietary choices and lifestyles.^1^ ^‐–^ ^3^ Nonalcoholic steatohepatitis (NASH) is characteristic of more than 5% hepatic steatosis and inflammation^4^ and at least 20% of NAFLD patients could progress to NASH,^4^ which might develop into chronic liver diseases like liver cirrhosis and cancer.^5^ According to the epidemiology from 2010 to 2019, NASH has become one of the main causes of liver cancer.^6^ Therefore, in order to settle this significant challenge of global health, it's urgent to find out the connection between metabolic disorders and uncontrollable inflammation.^2^

Mitochondrial dysfunction has been proven to regulate NASH progression by changing several metabolic pathways including hepatic glucose and lipid metabolism and oxidative stress.^7^ ^–‐^ ^9^ Moreover, mitochondria are not only dynamic biophysical systems in regulating metabolism but also harmonize different signalling pathways via the production of metabolite^2^, ^10^ which might dictate immunological fate.^11^ Inflammation is a characteristic of liver disease.^12^ ^–‐^ ^14^ Neutrophils are involved in fatty liver disease inflammation^15^ and are important players in NASH.^16^ However, how mitochondrial signals in the hepatocyte regulate neutrophils functions remains elusive.

Mitochondrial general control of amino acid synthesis 5 like‐1 (GCN5L1) functions in modulating protein acetylation in mitochondria, metabolism, mitochondrial turnover, autophagy, liver cancer and cardiac disease.^17^ ^–‐^ ^21^ Research has demonstrated the importance of GCN5L1 in the liver. For example, liver GCN5L1 knockout increases liver regenerative capacity.^22^ GCN5L1 controls hepatic glucose production^23^ and fatty acid oxidation enzymes.^24^ These works demonstrate the importance of GCN5L1 in the liver. Our previous works also find that GCN5L1 induces the progression of kidney disease by regulating lipid metabolism and oxidative metabolism.^25^, ^26^ Despite its central position in the liver and metabolism, its roles in NASH remain unclear.

In this research work, we illustrated that increased expression of GCN5L1 in NASH samples is connected with the severity of the disease. Hepatocyte‐specific GCN5L1 conditional knockout (GCN5L1 HKO) mice had reduced progression of NASH. However, overexpression of mitochondrial GCN5L1 (MtG) had the opposite effect. Mechanically, GCN5L1 promoted ferroptosis of hepatocytes by acetylating CypD and released mitochondrial ROS (mROS) via the opening of mitochondrial permeability transition pores (mPTPs). The dead hepatocyte released high mobility group box 1 (HMGB1) which recruited neutrophils and induced the generation of neutrophil extracellular traps (NETs), which promoted NASH progression. Inhibition of HMGB1 or NETs impaired GCN5L1‐induced NASH progression. Finally, we found that lipid‐induced endoplasmic reticulum (ER) stress contributed to the upregulation of GCN5L1 by ATF4. These works indicate that GCN5L1 is critical in promoting the progression of NASH.

## 2 MATERIALS AND METHODS

### 2.1 Human liver samples

The liver samples were collected from patients undergoing liver biopsy, liver surgery or liver transplantation. The exclusion criteria were described as follows: the male and female patients with drinking more than 140 or 70 g in a week respectively, using a drug or toxin with liver damage, viral infection or other liver disease were eliminated.^27^, ^28^ The study was authorized by the institutional ethics committee of Shandong provincial hospital and followed the Declaration of Helsinki. NAFLD activity score (NAS) following the roles: NAS ≥ 3 or 5 were sorted out as NAFLD and NASH respectively. Samples with NAS of 0 were treated as normal controls (NC).^29^

### 2.2 Animal studies

All animal studies were authorized by the Institutional Animal Care and Use Committee of Shandong provincial hospital. GCN5L1‐HKO mice were generated by crossing the GCN5L1^flox/flox^ mice and alb‐cre mice. Hepatic overexpression mitochondrial GCN5L1 was applied by adeno‐associated virus (AAV). All mice (C57BL/6 male) were fed in a standard environment with a temperature of 23 ± 2°C and 12 h/12 h light/dark cycle. Either normal chow (NC) or high‐fat/high‐cholesterol (HFHC) (60% energy from fat with 2.5% cholesterol) for 12 weeks^30^ or methionine‐choline deficient (MCD) from Trophic Diet, Nantong, China for 6 weeks was used to feed mice. Mice were treated with DNase (DNase I, Roche, 50 μg IP 3x/week)^29^ to inhibit neutrophil extracellular traps (NETs). 4‐PBA (an ER stress blocker, 1 g/kg/day supplemented in the drinking water)^31^ was applied in NASH models. After the experiment, body and liver weights were measured. Tissues and serum samples were collected for determining the level of triglycerides (TGs), total cholesterol, alanine transaminase and aspartate aminotransferase (AST) by using the Commercial kits.

### 2.3 Histological staining

The paraffin sections of liver tissues were stained with hematoxylin and eosin, Masson and Sirius Red staining. Frozen liver sections were used to stain with oil red O for visualizing the lipid accumulation. Masson and Sirius's Red staining were for evaluating the severity of fibrosis. Immunohistochemical (IHC) staining and immunofluorescence (IF) were described in the supplementary materials. Histological images were acquired under a microscope.

### 2.4 Transmission electron microscope

3% glutaraldehyde was first used to infiltrate liver tissues, and these tissues were embedded after the treatment of osmium tetroxide, dehydrated and Epox 812. Methylene was then used to handle semithin sections and ultrathin sections were processed by blue uranyl acetate and lead citrate. Samples were detected with JEM‐1400‐FLASH Transmission Electron Microscope (TEM).

### 2.5 Detection of ROS

Cell cytosolic ROS was monitored by DCFH‐DA (Cat# D6883, Sigma‐Aldrich) and mitochondrial ROS was detected by MitoSOX (Cat# M36008, Invitrogen) as described.^2^ In brief, 5 × 10^5^ cells/well were placed in a 6‐well cell culture plate and treated with indicated stimulus. After this, DCFH‐DA (10 mM) or MitoSOX (5 mM) working solution was used to deal with these cells at 37°C for 20 min avoiding light. Hoechst 33342 (1 mg/ml, Cat# H3570, Invitrogen) was used to stain nuclei. The liver tissue staining of ROS was detected by MitoSOX and dihydroethidium (DHE). Briefly, cryosections were incubated with 5 mM MitoSOX and 10 μM DHE at 37°C for 30 min. Finally, the fluorescence signal was detected by a fluorescence microscope system.

### 2.6 Lipid ROS measurement

Lipid ROS measurement was according to the procedure described in the study.^32^ Briefly, different isolated/treated hepatocyte or cultured cells were incubated with C11‐BODIPY working solution (50 μM, Thermo Fisher, Cat# D3861) for 1 h and the labelled cells were trypsinized and resuspended in PBS plus 5% FBS. Flow cytometry and IF were used to detect lipid ROS generation.

### 2.7 Detection of mPTP opening

Calcein‐AM loading/CoCl2 quenching was applied to assess mPTP opening through Transition Pore Assay Kit (Cat# I35103, Thermo Fisher Scientific).^2^ In brief, cells were incubated with 1 mM calcein‐AM and 5 mM cobalt chloride (CoCl2) at 37°C for 15 min in darkness. Calcein was excited at 494 nm, and emission was recorded at 517 nm. A fluorescence microscope was then used to evaluate the fluorescence signal.

More details about the methods and materials are available in Supporting Information.

## 3 RESULTS

### 3.1 GCN5L1 expression is increased in NASH patients and positively relates to the severity of NASH

**FIGURE 1:**
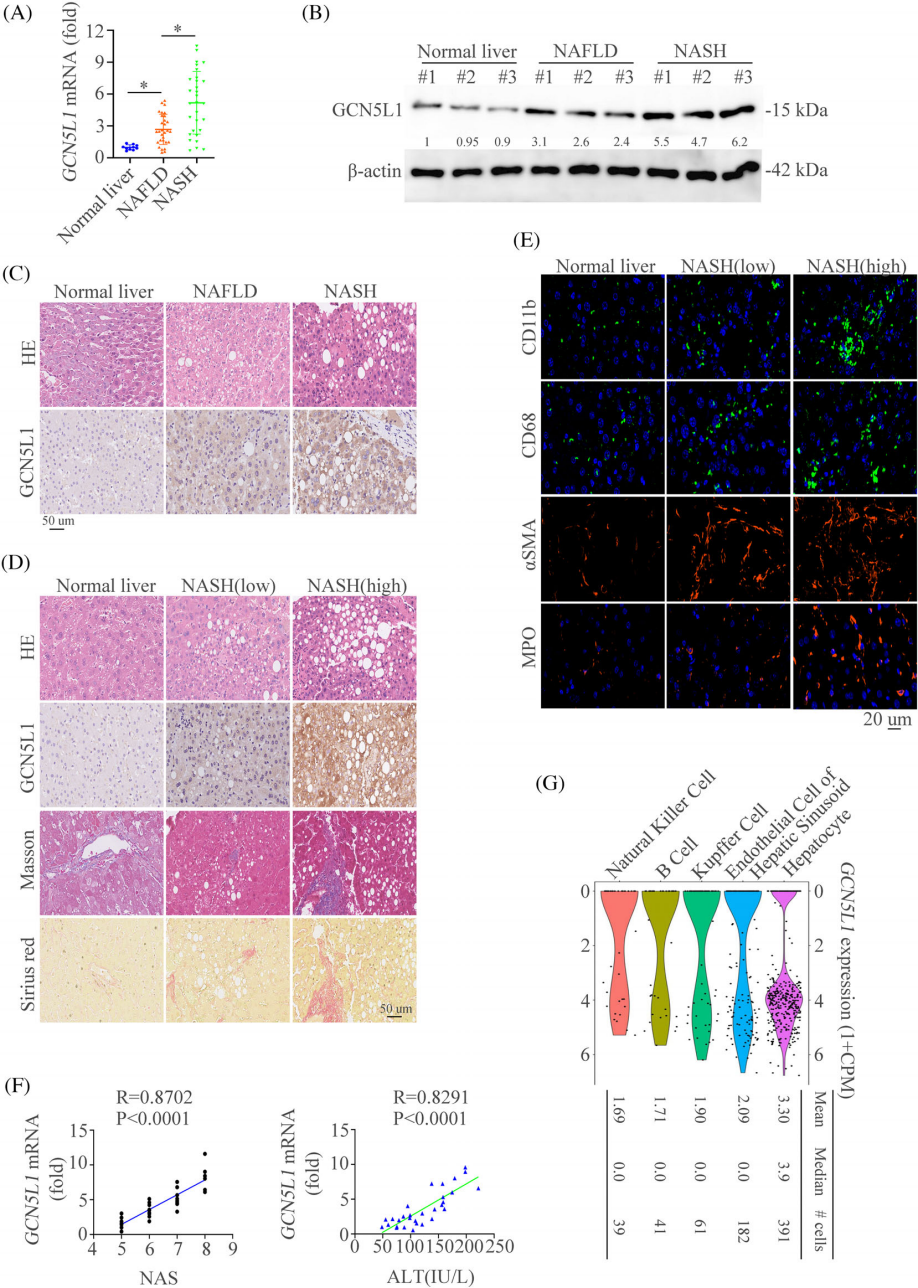
Hepatic expression of GCN5L1 is upregulated in NASH patients and positively correlates with disease severity (A) GCN5L1 mRNA levels in liver tissues of human normal controls (n = 10), non‐alcoholic fatty liver disease (NAFLD) (n = 30), and NASH (n = 30) as determined by RT‐qPCR. (B) Western blot was used to show the expression levels of GCN5L1 protein in liver extracts from patients with normal controls, NAFLD and NASH. (C) Representative Hematoxylin and Eosin (H&E) and immunohistochemistry (IHC) images of control, NAFLD, and NASH liver sections were shown. Scale bars indicate 50 μm. (D) H&E, GCN5L1 expression by IHC, Masson staining and Sirius Red in liver sections from normal controls, NAFLD and NASH. Scale bars indicate 50 μm. (E) Representative immunofluorescence (IF) staining showed CD11b, CD68, α‐SMA and myeloperoxidase (MPO). Scale bars indicate 20 μm. (F) The correlation between *GCN5L1* mRNA levels and NAS and serum alanine transaminase (ALT) levels in patients with NASH. (G) Expression profiles of GCN5L1 in mice liver. Error bars represent mean ± SD. *p < 0.05.

In order to detect the importance of GCN5L1 in NAFLD and NASH, the expression of GCN5L1 was first analyzed in liver samples from control, NAFLD and NASH patients. Both the mRNA and protein levels of GCN5L1 were higher in NASH liver samples than in NAFLD and normal liver tissues (Figure 1A,B). The IHC staining of GCN5L1 also proved these results (Figure 1C). The clinical importance of GCN5L1 was also evaluated. We sorted the NASH patients into two groups according to the median value of GCN5L1 tested by RT‐qPCR: GCN5L1 high expression (15/30) and GCN5L1 low expression (15/30). According to the histological staining, we found that the liver tissues with higher GCN5L1 expression had more hepatic lipid accumulation, worse fibrosis, more infiltration of inflammatory cells, and more serious liver damage (Figure 1D‐‐F, Figure S1A). To find out the abundance of GCN5L1 in different liver cells, the Tabula Muris database^33^ was used. Single‐cell sequencing of mice tissues found that GCN5L1 was mainly located in hepatocytes among the top five liver cell types (Figure 1G). Overall, all data demonstrate the importance of GCN5L1 in the progression of NASH.

### 3.2 Upregulated GCN5L1 level is shown in mouse NASH models and hepatic GCN5L1 deficiency improves NASH progression

**FIGURE 2:**
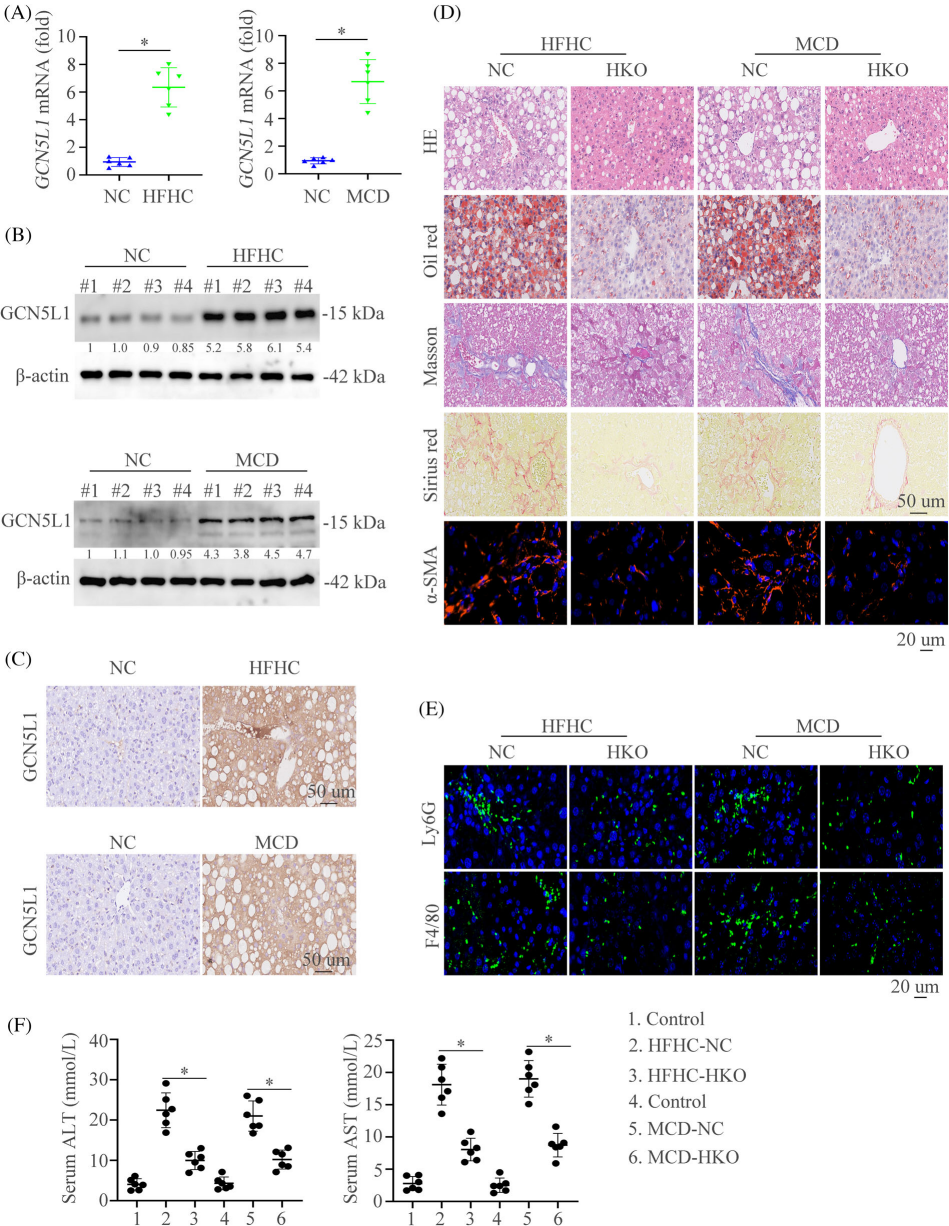
Upregulated GCN5L1 level is shown in mouse NASH models and hepatic GCN5L1 deficiency ameliorates diet‐induced NASH (A) Hepatic mRNA in wildtype (WT) mice fed the control, HFHC, or MCD diet. (B) Protein levels in wildtype (WT) mice fed the control, HFHC, or MCD diet. (C) Immunohistochemical (IHC) showed the GCN5L1 expression in indicated mice groups. (D) Hematoxylin and eosin (H&E), Oil Red O, Masson, Sirius Red, and α‐SMA staining were shown in WT or GCN5L1 HKO mice fed with HFHC, or MCD diet. (E) Representative immunofluorescence (IF) staining showed Ly6G and F4/80. (F) Graphs showed the serum alanine transaminase (ALT) and aspartate aminotransferase (AST) in mice (n = 6/group). Scale bars indicate 50 μm for IHC and 20 μm for IF. Error bars represent mean ± SD. *p < 0.05.

In order to detect the expression of GCN5L1 in mouse NASH models, we first established two mouse models and we found that hepatic GCN5L1 expression was increased in HFHC or MCD diets‐induced NASH mouse models (Figure 2A‐‐C). We then established the special HKO mice of GCN5L1 (GCN5L1 HKO) (Figure S1B). 8‐week‐old GCN5L1^flox/flox^ and GCN5L1 HKO mice were fed with HFHC or MCD diet to gain the NASH models. Histological staining including Hematoxylin and Eosin (HE), Oil Red O, Masson, Sirius Red, and IF staining of αSMA were used to evaluate the impact of GCN5L1 in NASH. The results found that GCN5L1 deficiency in hepatocytes ameliorated hepatic inflammation, lipid accumulation, and fibrosis (Figure 2D). We then used the IF to detect the number of macrophages and neutrophils and found reduced numbers of these immune cells in GCN5L1 HKO mice compared to GCN5L1^flox/flox^ mice as detected by IF (Figure 2E). Furthermore, GCN5L1 deficiency in consistently decreased liver enzymes, which indicated the protective functions of GCN5L1 deficiency (Figure 2F). Liver weight, TG and cholesterol levels were decreased in GCN5L1 deficiency mice (Figure S1C–G). Indicators of cell damage like PCNA and γH_2_AX were also decreased in GCN5L1 deficiency mice (Figure S1H,I). These results demonstrate that GCN5L1 HKO plays a protective role in NASH progression.

### 3.3 Hepatocyte‐specific overexpression of mitochondrial GCN5L1 aggravates NASH

**FIGURE 3:**
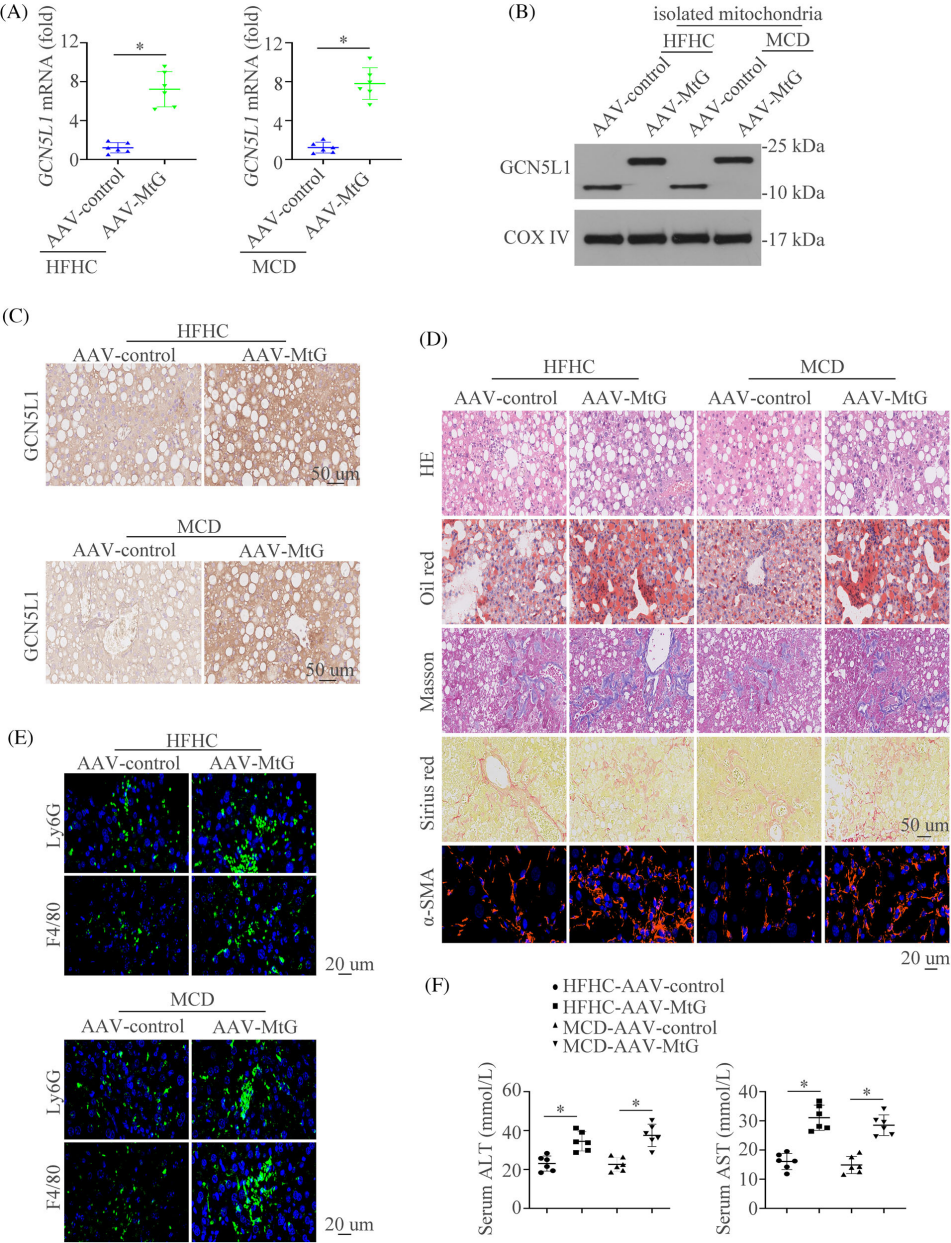
Hepatocyte‐specific overexpression of mitochondrial GCN5L1 aggravates NASH (A‐C) Hepatic mRNA (A), protein levels (B) and immunohistochemical (IHC) staining of GCN5L1 (C) in AAV‐control or AAV‐MtG mice fed with HFHC, or MCD diet. (D) Hematoxylin and eosin (H&E), Oil Red O, Masson, Sirius Red, and α‐SMA staining were shown in AAV‐control or AAV‐MtG mice fed with HFHC, or MCD diet. (E) Representative immunofluorescence (IF) staining showed Ly6G and F4/80. (F) Graphs showed the serum alanine transaminase (ALT) and aspartate aminotransferase (AST) in mice (n = 6/group). Scale bars indicate 50 μm for IHC and 20 μm for IF. Error bars represent mean ± SD. *p < 0.05.

To further find out the importance of GCN5L1 in NASH in *vivo*, we constructed a mitochondrial overexpression of GCN5L1 (MtG) mouse model by applying the AAV (AAV‐MtG). The expression and location of MtG were confirmed by RT‐qPCR, western blot, IHC and IF in NASH models (Figure 3A‐‐C and Figure S2A). Mitochondrial‐restricted GCN5L1 overexpression and littermate mice were subjected to feed with NC or HFD diet. HE, Oil Red O, Masson, Sirius Red, and IF staining of αSMA illustrated that GCN5L1 overexpression aggravated liver inflammation, lipid accumulation, and fibrosis (Figure 3D). Meanwhile, increased infiltration of macrophages and neutrophils was shown in AAV‐MtG mice compared to AAV‐control mice as detected by IF (Figure 3E). Furthermore, mice with GCN5L1 upregulation had increased liver enzymes (Figure 3F). Liver weight, TG and cholesterol levels were largely increased in GCN5L1 upregulation mice (Figure S2B–D). The IHC staining of γH_2_AX and PCNA was stronger in AAV‐MtG mice than in controls (Figure S2E,F). These data illustrate the importance of hepatic GCN5L1 in liver inflammation and liver damage in NASH mouse models.

### 3.4 GCN5L1 promotes ferroptosis of hepatocytes during NASH progression

**FIGURE 4:**
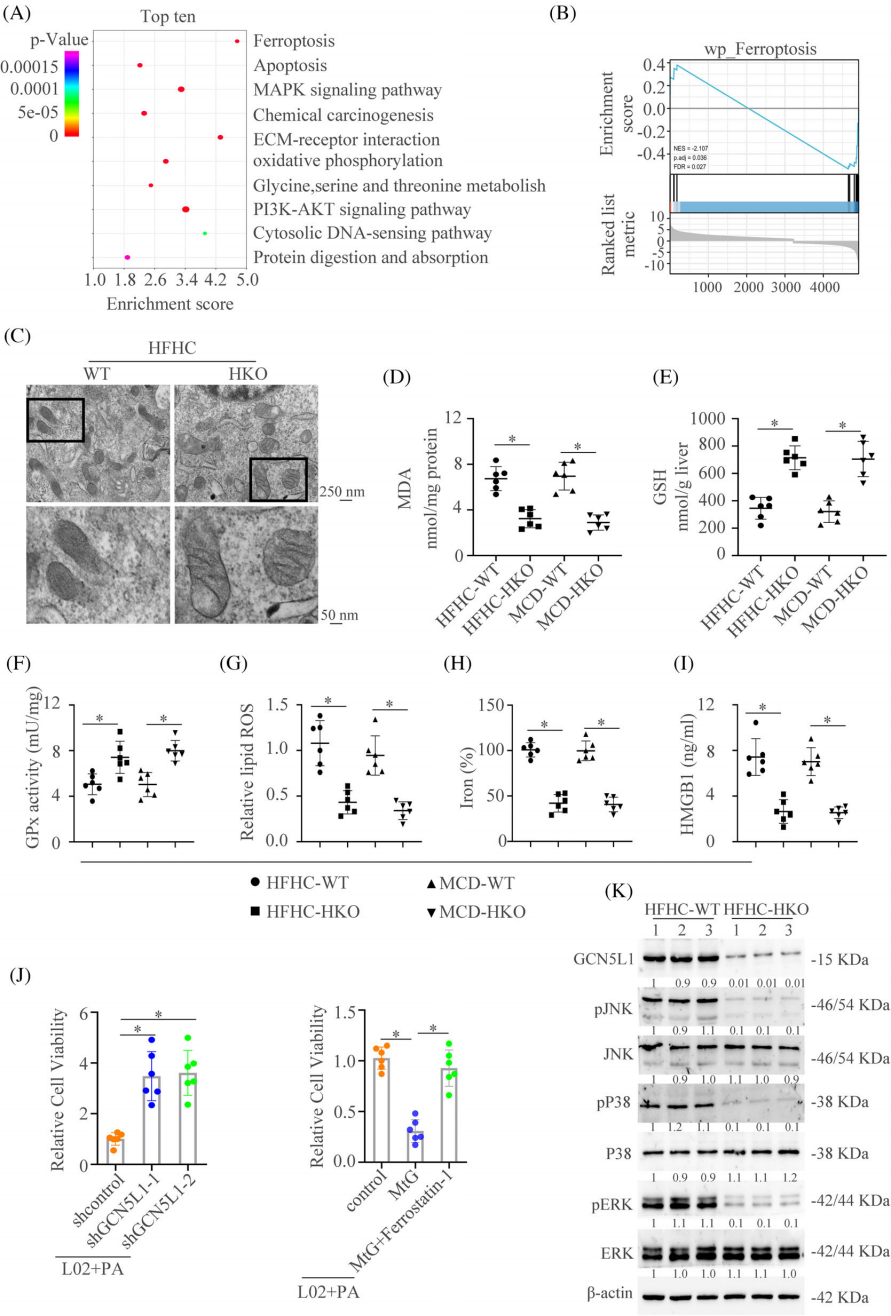
GCN5L1 promotes ferroptosis of hepatocyte during NASH progression (A) KEGG analysis showed the top 10 pathway enrichment in GCN5L1 HKO group versus WT group. (B) Gene Set Enrichment Analysis (GSEA) showed the importance of ferroptosis in the GCN5L1 HKO group versus the WT group. (C) Transmission electron microscope showed the mitochondria in liver tissues. (D–F) Malondialdehyde (MDA), GSH, and GPx activity were assayed in WT and GCN5L1 HKO mice treated with HFHC or MCD. (G) Lipid ROS from isolated hepatocytes was measured by C11‐BODIPY staining coupled with flow cytometry. (H) Iron content was measured in WT and GCN5L1 HKO mice treated with HFHC or MCD. (I) HMGB1 was measured by ELISA in the serum of indicated groups. (J) Cell viability was assayed in different indicated cells. (K) Western blot was applied to detect the activation of the MAPK pathway.

In order to detect the mechanism of GCN5L1 in regulating NASH progression, RNA sequencing analysis was performed by using the liver tissues of HFHC‐WT and HFHC‐HKO. The Kyoto Encyclopedia of Genes and Genomes (KEGG) pathway analysis identified the top 10 changed pathways between HFHC‐WT and HFHC‐HKO. The ferroptosis pathway was the most significant changed axis in these pathways (Figure 4A). Gene Set Enrichment Analysis also identified the importance of ferroptosis in regulating GCN5L1‐induced NASH (Figure 4B).

To prove the roles of ferroptosis in GCN5L1‐regulated NASH, TEM was used to detect the morphological changes of mitochondria in liver tissues. The mitochondria in liver tissues of GCN5L1‐WT exhibited a characteristic morphologic feature of ferroptosis: shrunken mitochondria with increased membrane density.^34^ However, GCN5L1‐HKO reversed these features (Figure 4C). GCN5L1 HKO downregulated the level of malondialdehyde (MDA), which is the end product of lipid peroxidation^35^ (Figure 4D). Moreover, GCN5L1 HKO decreased ferroptotic events, including increased GSH level and GPx activity (Figure 4E,F), and decreased lipid ROS production and iron content (Figure 4G,H). Ferroptotic cell death released the HMGB1 into the extracellular environment.^36^, ^37^ Therefore, we then detected the HMGB1 level in mice. The results showed that GCN5L1 HKO could decrease the HMGB1 release in both NASH models (Figure 4I). To further demonstrate the importance of ferroptosis in GCN5L1‐regulated NASH progression, we used the L02 cell line to prove these results. We found that increased cell viability, decreased lipid ROS and MDA levels and enhanced GSH were shown under palmitic acid (PA) treatment with GCN5L1 knockdown (Figure 4J left, Figure S3A–C). GCN5L1 overexpression decreased cell viability and this effect could be reversed by a ferroptotic inhibitor ferrostatin‐1 (Figure 4J right). Similarly, increased lipid ROS and MDA, and decreased GSH could be reversed by ferrostatin‐1 (Figure S3D–F). The role of GCN5L1 in ferroptosis was also confirmed by the L02 treated with the ferroptosis inducer erastin (Figure S3G–I). MAPK pathway has been proven to participate in the process of ferroptosis.^38^ Therefore, we then detected the changes in the MAPK pathway in the NASH model. We found that GCN5L1 HKO could decrease the activation of the MAPK pathway (Figure 4K). Collectively, these results illustrate that GCN5L1 could regulate ferroptosis during NASH progression.

### 3.5 GCN5L1 directly binds to CypD and promotes the acetylation of CypD

**FIGURE 5:**
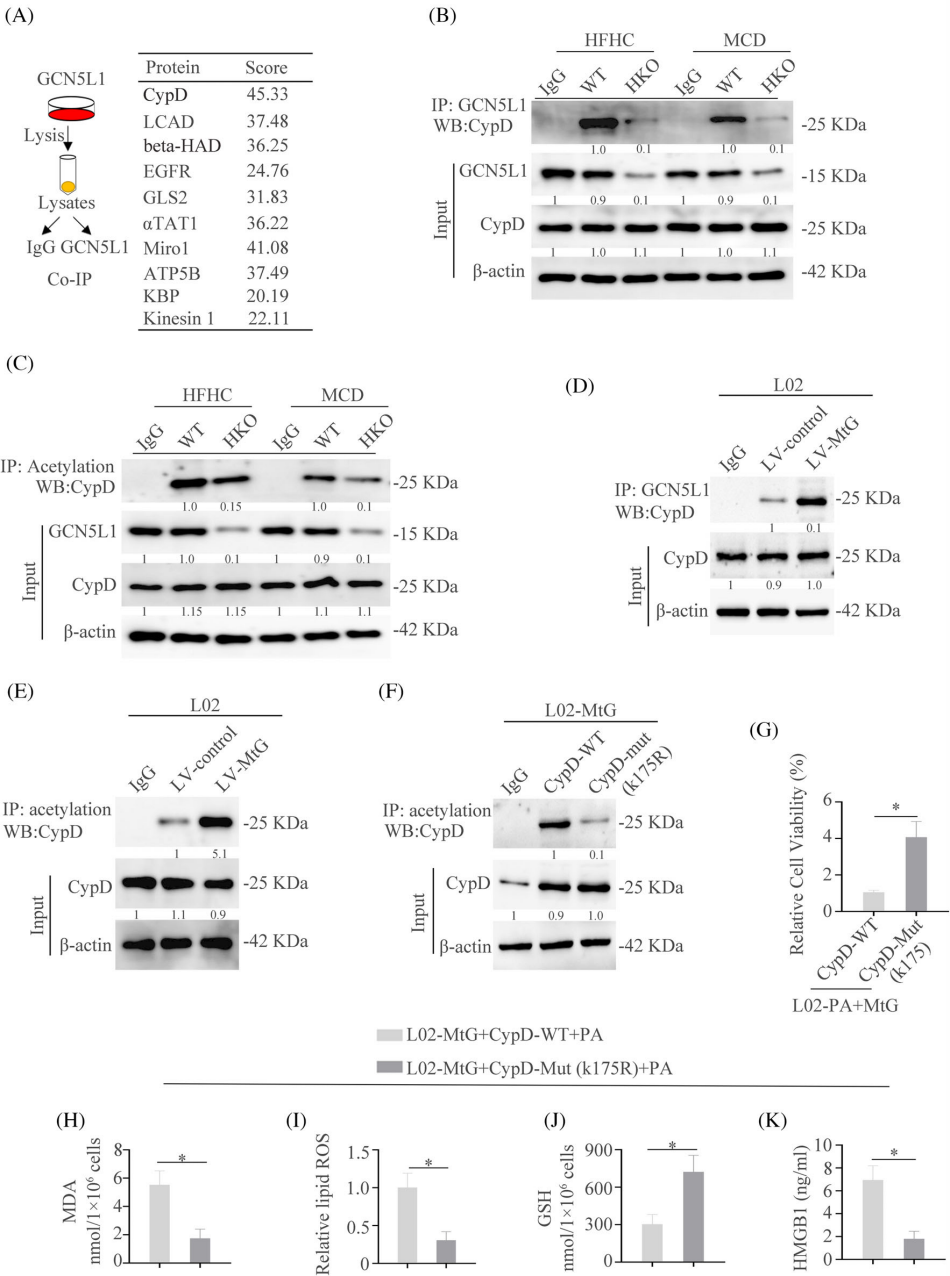
GCN5L1 directly binds to CypD and promotes the acetylation of CypD (A) Top 8 proteins were screened by the protein mass spectrometry and their potential interactions. (B) Co‐IP was used to show the interaction of GCN5L1 and CypD in WT and GCN5L1 HKO mice fed with HFHC or MCD. (C) IP was used to show the acetylation of CypD in WT and GCN5L1 HKO mice fed with HFHC or MCD. (D) Co‐IP was used to show the interaction of GCN5L1 and CypD in L02 cells transfected with control or MtG. (E) IP was used to show the acetylation of CypD in L02 cells transfected with control and MtG. (F) IP was used to show the acetylation of CypD in L02‐MtG cells transfected with WT or a mutation of CypD. (G) Cell viability was assayed in different indicated cells. (H–J) Malondialdehyde (MDA), lipid ROS and GSH were assayed in L02‐MtG cells with PA and transfected with WT or mutation of CypD. (K) HMGB1 was measured by ELISA in the serum of indicated groups.

Next, we investigated the mechanism of GCN5L1‐induced ferroptosis. The top 8 proteins and their potential interactions were presented in Figure 5A and CypD ranked first among them. Co‐IP and western blot verified the interaction between GCN5L1 and CypD in NASH tissues and the knockout of GCN5L1 impaired the interaction (Figure 5B). Moreover, we found that acetylation of CypD was increased in HFHC tissues and GCN5L1 knockout decreased the acetylation of CypD (Figure 5C). In order to further identify these results in vitro, we upregulated the expression of GCN5L1 in L02 cells and the results illustrated that GCN5L1 could increase the binds of CypD and promote acetylation of CypD (Figure 5D,E). Furthermore, three amino acid sites were identified to be acetylated by GPS‐PAIL^39^ (Figure S4A) and the analysis of the mutation site showed only the lysine K175 as the acetylation site on CypD targeted by GCN5L1. K175 mutation decreased the acetylation of CypD caused by GCN5L1 (Figure 5F and Figure S4B,C). K175 mutation could increase the cell viability caused by GCN5L1 upregulation (Figure 5G). Moreover, K175 mutation decreased ferroptotic events driven by GCN5L1 upregulation, including the depletion of MDA and lipid ROS (Figure 5H,I and Figure S4D), increased GSH content (Figure 5J) and impaired HMGB1 release (Figure 5K). All data illustrate that GCN5L1 could acetylate CypD at the K175 site.

### 3.6 GCN5L1 promotes mPTP opening and mitochondrial ROS output by inducing interaction between CypD and ATP5B

**FIGURE 6:**
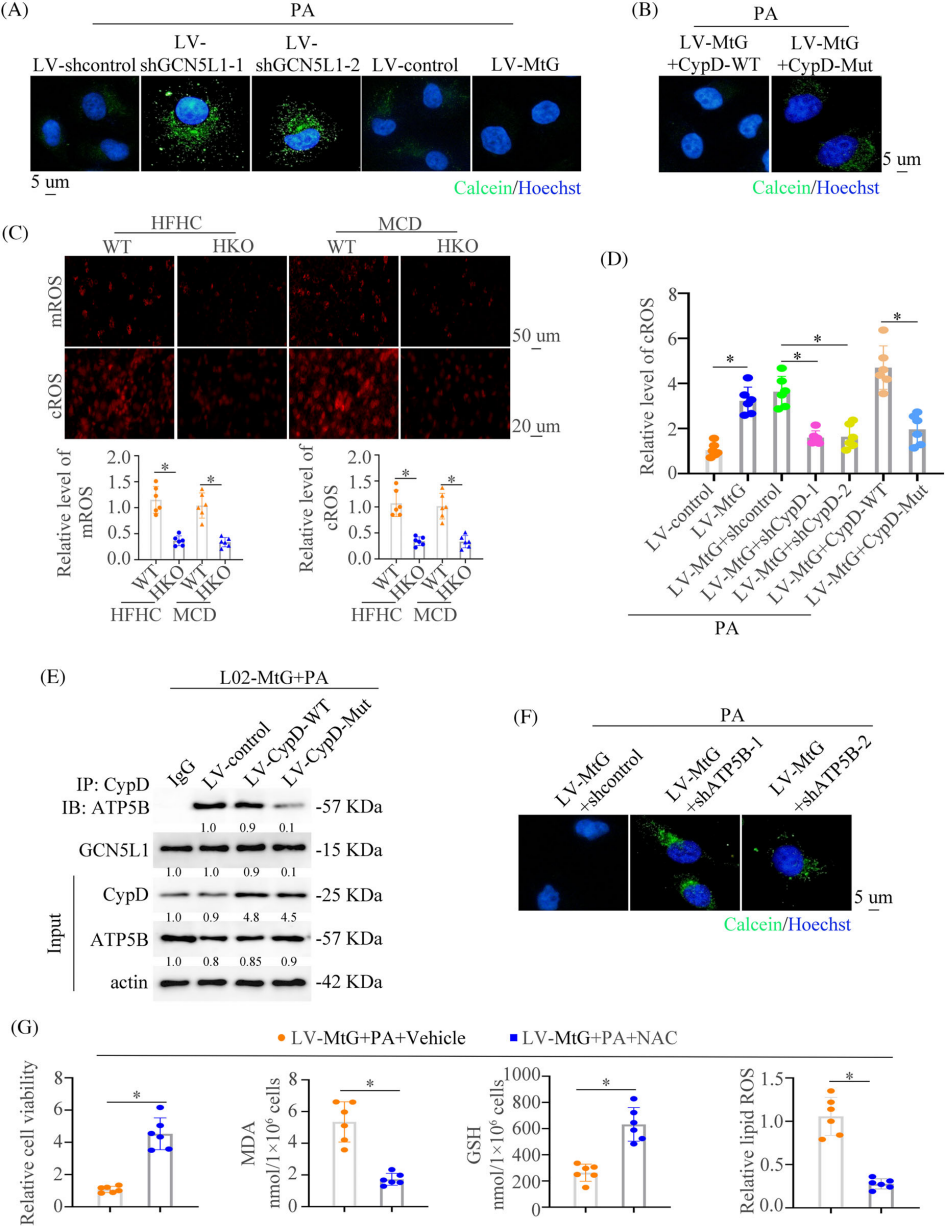
GCN5L1 promotes mPTP opening and mitochondrial ROS output by inducing interaction between CypD and ATP5B (A) The mPTP opening was measured by calcein loading in L02 cells transfected with LV‐shcontrol, LV‐shGCN5L1, LV‐control or LV‐MtG under PA treatment. (B) The mPTP opening was measured by calcein loading quenching in L02‐MtG cells transfected with CypD‐WT or mutation of CypD. (C) Representative images of MitoSOX (up) and DCFH‐DA (down) staining in liver tissues of NASH mice. (D) Cytoplasmic ROS staining in cells with indicated treatment. (E) Co‐IP was used to show the interaction of ATP5B and CypD in indicated cells. (F) The mPTP opening was measured by calcein loading in L02‐MtG cells transfected with shcontrol or shATP5B under PA treatment. (G) Cell viability, malondialdehyde (MDA), GSH, and lipid ROS were assayed in L02 cells transfected with LV‐MtG with or without NAC supplement.

CypD is the main regulator in inducing the opening of mPTP, which increases mROS generation and release.^40^ To evaluate whether GCN5L1 promotes mPTP opening, a calcein release experiment was performed. PA resulted in a large decrease in calcein fluorescence, manifesting mPTP opening and GCN5L1 knockdown resulted in an increase in calcein fluorescence caused by PA. Meanwhile, upregulation of GCN5L1 aggravated mPTP opening induced by PA with a lower fluorescence (Figure 6A). However, CypD mutation reversed mPTP opening (Figure 6B). These results were confirmed by flow cytometry (Figure S4E). These results demonstrated that GCN5L1 might induce the opening of mPTP. The mROS staining also demonstrated that high lipid could induce mROS output and GCN5L1 knockdown decreased the mROS and cytoplasmic ROS (cROS) staining (Figure 6C). The knockdown and mutation of K175 of CypD reversed the cROS output induced by GCN5L1(Figure 6D).

Studies have illustrated that CypD could bind to ATP5B and regulate the mPTP opening.^41^, ^42^ Therefore, we asked whether GCN5L1‐regulated CypD acetylation disrupted CypD‐ATP5B interaction. The co‐IP experiment found that GCN5L1 largely augmented the binding of CypD to ATP5B, which was blocked by a mutant of CypD (Figure 6E). Furthermore, we found that ATP5B knockdown could impair the mPTP opening driven by GCN5L1 (Figure 6F). The above works showed that GCN5L1 induced mROS output by regulating mPTP opening. Therefore, we hypothesized that the increased ROS in the cytoplasm could induce ferroptosis. mROS block by MitoQ increased the cell viability and decreased ferroptotic events, including the depletion of MDA and lipid ROS, increased cell viability and GSH content driven by GCN5L1 upregulation (Figure S4F). The removal of ROS by NAC increased the cell viability and GSH level and decreased the content of MDA and lipid ROS (Figure 6G and Figure S4G). NAC treatment had little effect on the necroptosis (Figure S4H). All of these results demonstrate that GCN5L1 acetylates CypD and promotes the binding to ATP5B, which opens the mPTP. The opening of mPTP induces the increase of cROS and promotes the ferroptosis of hepatocytes.

### 3.7 GCN5L1 promotes the recruitment of neutrophils and formation of neutrophil extracellular traps by the ferroptosis‐induced release of HMGB1

**FIGURE 7:**
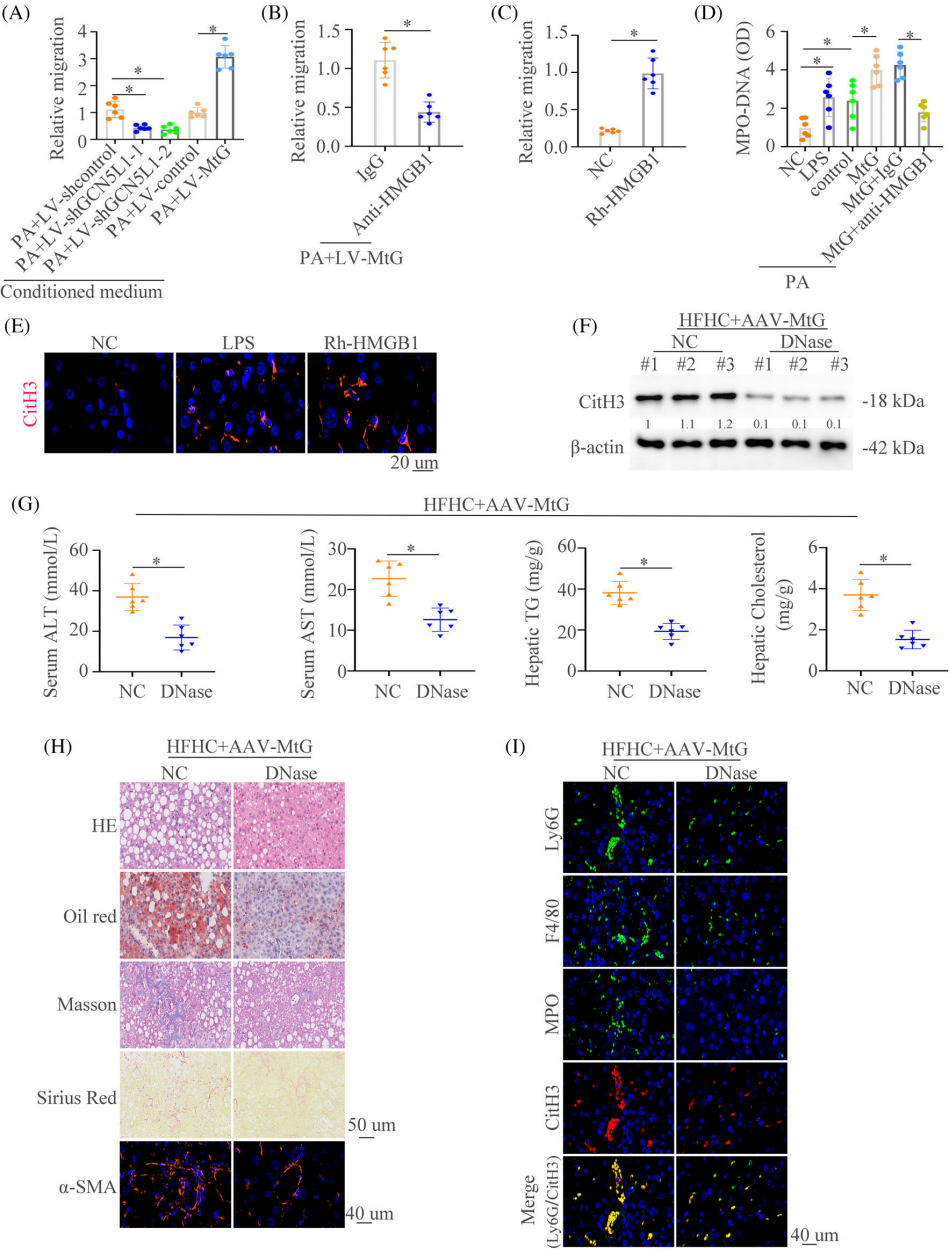
GCN5L1 promotes the recruitment of neutrophils and formation of neutrophil extracellular traps by ferroptosis‐induced release of HMGB1 (A,B) Neutrophils were treated with conditioned medium from the indicated cells. The migrative ability of neutrophils was detected in different groups. (C) Neutrophils were treated with NC or Rh‐HMGB1 and The migrative ability of neutrophils was detected. (D) MPO‐DNA levels were measured in different groups. (E) Representative immunofluorescence (IF) images of CitH3 staining were shown. (F) Western blot of liver tissues from HFHC mice with special hepatocyte overexpression of AAV‐MtG with or without DNase showed the expression of citrullinated histone 3 (CitH3). (G) Graphs showed the serum alanine transaminase (ALT), aspartate aminotransferase (AST), hepatic triglyceride (TG) and cholesterol in mice (n = 6/group). (H) Hematoxylin and eosin (H&E), Oil Red O, Masson, Sirius Red, and α‐SMA staining were shown in AAV‐ AAV‐MtG NASH mice with or without DNase. (I) Representative IF staining showed Ly6G, F4/80, MPO and CitH3 in AAV‐MtG NASH mice with or without DNase. Scale bars indicate 50 μm for immunohistochemical (IHC) and 40 μm for IF. Error bars represent mean ± SD. *p < 0.05.

Extracellular HMGB1 usually regulates inflammation and the immune microenvironment.^43^, ^44^ Our above works have demonstrated that GCN5L1 induced the infiltration of neutrophils (Figure 2E) and the release of HMGB1 by ferroptosis (Figure 4I). Therefore, we conjectured that GCN5L1 might recruit neutrophils by HMGB1. The results demonstrated that the knockdown of GCN5L1 could decrease the neutrophils migration and upregulation of GCN5L1 reverse the migration (Figure 7A). However, the migration of neutrophils triggered by MtG could be reversed by anti‐HMGB1 treatment (Figure 7B). The treatment of RhHMGB1 strengthened the migration of neutrophils (Figure 7C). Neutrophil extracellular traps (NETs) aggravate NASH.^45^ We next detected whether GCN5L1/HMGB1 pathway could regulate the formation of NETs. By measuring NETs‐specific marker myeloperoxidase (MPO) associated with DNA (MPO‐DNA) and CitH3,^46^ we can be certain that GCN5L1 treatment increased the contents of MPO‐DNA and CitH3. Anti‐HMGB1 could decrease the levels of MPO‐DNA and impair the expression of CitH3 (Figure 7D and Figure S5A). IF and western blot analysis revealed that CitH3 was present under the HMGB1 treatment (Figure 7E,F). All of these works demonstrated that the GCN5L1‐HMGB1 axis is important in NETs formation.

After confirming that GCN5L1 could promote the formation of NETs, we tried to determine whether the GCN5L1‐NETs axis contributed to the progression of NASH. We treated the NASH models with NETs inhibitor DNase. The results showed that DNase could decrease liver enzymes, TG and cholesterol levels in HFHC groups and in HFHC groups with GCN5L1 upregulation (Figure 7G and Figure S5B). HE, Oil Red O, Masson, Sirius Red, IF staining of αSMA and NAS score found that DNase decreased liver inflammation, lipid accumulation, and fibrosis (Figure 7H and Figure S5C). Meanwhile, decreased infiltration of macrophages and neutrophils was observed in DNase‐treated mice compared to controls (Figure 7I). These data illustrate that NETs inhibition could improve the NASH progression driven by the upregulation of GCN5L1.

### 3.8 Lipid‐induced ER stress contributes to the upregulation of GCN5L1 in NASH

**FIGURE 8:**
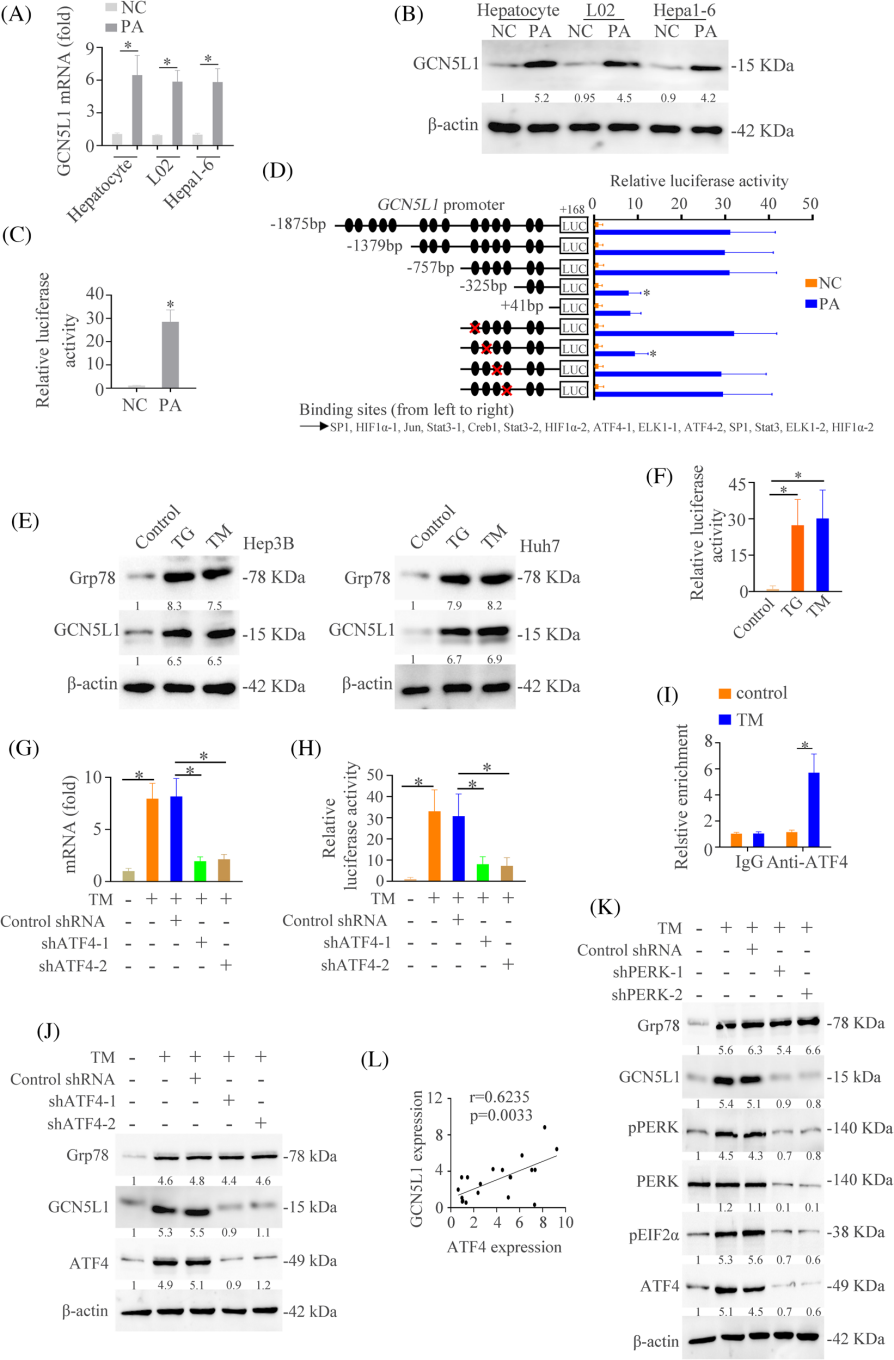
Lipid‐induced endoplasmic reticulum (ER) stress contributes to the upregulation of GCN5L1 in NASH (A) The mRNA expression of *GCN5L1* in the L02 cells with or without PA treatment. (B) The protein level of GCN5L1 in the L02 cells with or without PA treatment. (C) Luciferase activity of *GCN5L1* mRNA promoter was detected in the L02 cells with or without PA treatment. (D) Truncated and mutated *GCN5L1* promoter sequences were established and transfected in L02 cells under PA treatment. Luciferase activity was detected. (E) Western blot analyzed the expression of GCN5L1 and β‐actin in indicated cells. (F) Luciferase activity of *GCN5L1* mRNA promoter was detected in the L02 cells with triglyceride (TG) or TM treatment. (G) The mRNA expression of *GCN5L1* in the L02 cells transfected with shATF4 under the TM treatment. (H) Luciferase activity of *GCN5L1* mRNA promoter was detected in the L02 cells transfected with shATF4 under the TM treatment. (I) Ch‐IP analysis was used to detect the binding of ATF4 and GCN5L1 promoter in the L02 cells with TG or TM treatment. (J) Western blot analyzed the expression of Grp78, GCN5L1, ATF4 and β‐actin in indicated HCC cells. (K) Protein expression of GCN5L1 and activation of ER stress in indicated HCC cells were detected. (L) The correlation between GCN5L1 and ATF4 levels in patients with NASH.

Our above works found that GCN5L1 was upregulated in NASH. Therefore, we hypothesized that lipids might be a contributor to GCN5L1 upregulation. RT‐qPCR and western blot demonstrated that PA could upregulate GCN5L1 expression (Figure 8A,B). Moreover, PA treatment increased the promoter activity of *GCN5L1* (Figure 8C). To find out which cis‐regulatory elements of the *GCN5L1* promoter had a function in PA treatment, a series of truncated mutants of the *GCN5L1* promoter were constructed. A deletion from nt −757 to −325 greatly lowed *GCN5L1* promoter activity caused by PA, hinting that this sequence was vital for the activation of the *GCN5L1* promoter induced by PA. Four transcription factor binding sites were found in this part. Mutation of these sites showed that ATF4 mutation in the binding site decreased *GCN5L1* promoter activity caused by PA (Figure 8D). ATF4 inhibition could decrease the PA‐induced GCN5L1 expression (Figure S5D).

ATF4 is a well‐known transcription factor of endoplasmic reticulum (ER) stress downstream, which is vital in the progression of NASH.^47^, ^48^ Therefore, we assessed whether ER stress was involved in GCN5L1‐induced NASH. We first measured the level of the ER stress marker Grp78 in NASH models and control models and showed that Grp78 was increased in NASH models (Figure S5E). We treated the L02 cells with thapsigargin (TG) and tunicamycin (TM) and the results showed that GCN5L1 expression was upregulated by TG and TM (Figure 8E). Moreover, TG and TM treatment increased the promoter activity of *GCN5L1* (Figure 8F). We next evaluated whether ER stress‐ATF4 pathway medicated GCN5L1 expression. RT‐qPCR and luciferase activity analysis demonstrated that the knockdown of ATF4 decreased the mRNA level of *GCN5L1* and impaired the *GCN5L1* promoter activity regulated by TM (Figure 8G,H). A ChIP test showed that TM increased the binding of ATF4 to the *GCN5L1* promoter (Figure 8I). Western blot demonstrated that ATF4 knockdown decreased TM‐induced GCN5L1 expression (Figure 8J). PERK is the upstream of ATF4.^47^ The western blot demonstrated that PERK knockdown decreased TM‐induced ATF4 and GCN5L1 expression (Figure 8K). Co‐expression analysis also found the positive relativity between GCN5L1 and ATF4 (Figure 8L). Finally, we established the NASH models with an MCD diet and treated these mice with or without ER stress inhibitor 4‐PBA. The results found that 4‐PBA decreased the GCN5L1 expression and NASH progression (Figure S5F,G). These studies suggested that lipid upregulated GCN5L1 expression through ER stress pathway.

## 4 DISCUSSION

**FIGURE 9:**
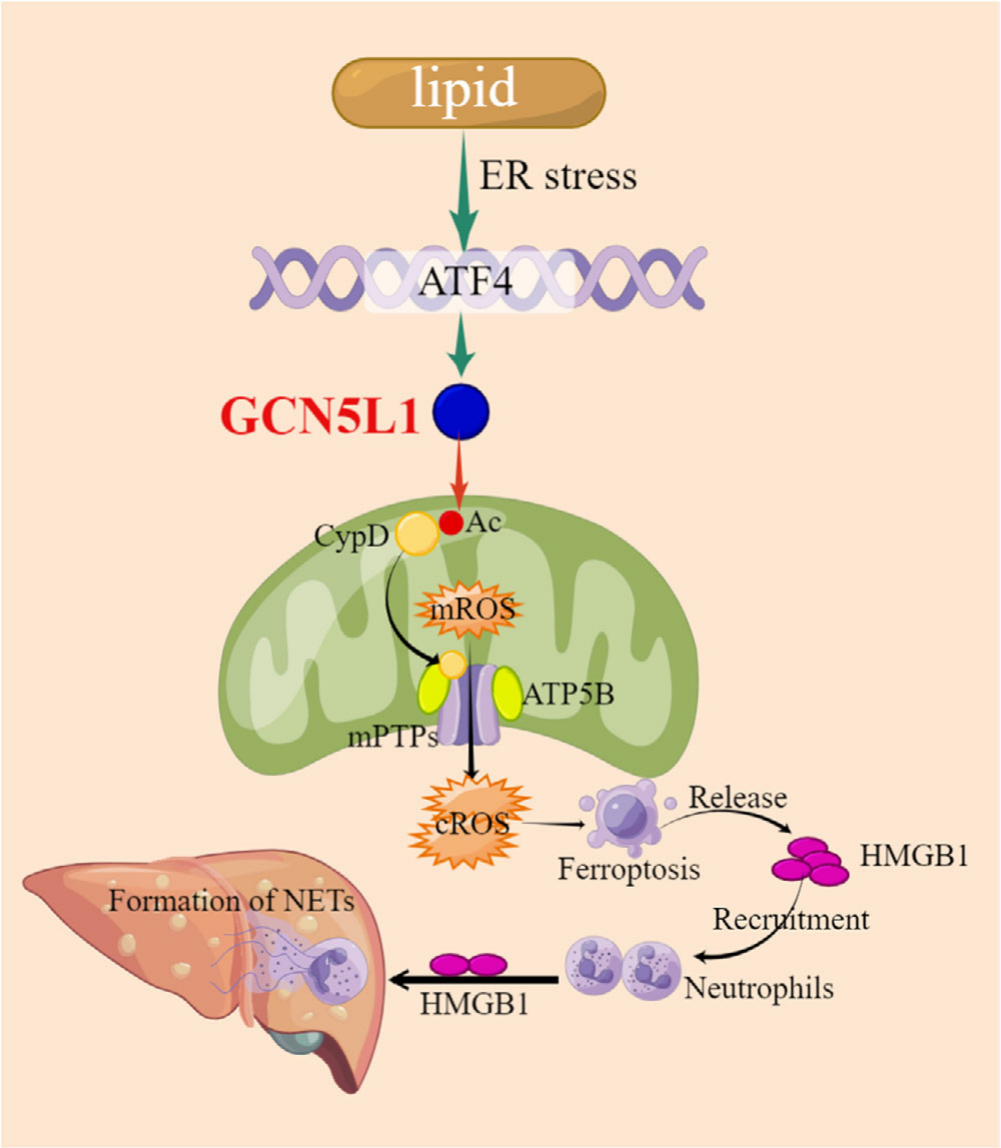
Graphical Abstract GCN5L1 acetylated CypD and released mitochondrial ROS (mROS) via the opening of mitochondrial permeability transition pores (mPTPs). The dead hepatocyte induced the release of high mobility group box 1 (HMGB1) which recruited neutrophils and induced the formation of neutrophil extracellular traps (NETs), which promoted NASH progression. Lipid‐induced endoplasmic reticulum (ER) stress contributed to the upregulation of GCN5L1 by ATF4.

NAFLD is now recognized as one of the most common liver diseases worldwide and is estimated at ∼25% of the population.^49^ NASH, a progressive type of NAFLD, has become one of the main sources of liver cirrhosis and liver cancer.^50^ Although the substantial burden of ill health is caused by NAFLD, the therapeutic strategies are limited.^3^ Therefore, the requirement on exploring the mechanistic investigation is urgent. In this research, we found out the role of GCN5L1 in NASH. Upregulation of GCN5L1 indicated the severity of NASH. The knockout of GCN5L1 in hepatocytes improved NASH. However, the specific overexpression of GCN5L1 in hepatocytes aggravates NASH. Mechanically, GCN5L1 induced ferroptosis of hepatocytes by acetylating CypD, which promoted the interaction of CypD and ATP5B and leaked the mROS. The ferroptosis‐induced release of HMGB1 recruited the neutrophils and promoted the formation of NETs. Furthermore, we found that lipid‐ER stress upregulated the expression of GCN5L1 through ATF4 (Figure 9).

GCN5L1 is located in mitochondria and induces acetylation of mitochondrial proteins, which is dependent on acetyl‐CoA production^51^ and regulates mitochondrial metabolism.^17^ Although several studies have found the importance in the liver such as regulating liver regeneration^22^ and controlling hepatic glucose production,^23^ the roles of GCN5L1 in NASH are unclear. In this study, we found the upregulation of GCN5L1 in NASH and indicated the progression of NASH. GCN5L1 induced the formation of liver inflammation, fibrosis and inflammatory infiltration. These results indicated the importance of GCN5L1 in NASH.

Hepatocyte exposure to excessive lipids causes oxidative stress and cell damage.^52^ Oxidative stress leads to cellular dysfunction and is considered a causative factor in the pathophysiology of NAFLD by inducing cellular lipotoxicity, lipid peroxidation, ER stress, and mitochondrial dysfunction.^53^ However, how mitochondrial ROS signalling connected the NASH is largely unknown. mPTP opening plays a significant role in maintaining mitochondria homeostasis and regulating mROS release.^54^ In this work, we figured out a novel function of GCN5L1 in regulating mPTP opening. GCN5L1 acetylated CypD and promoted interaction between CypD and ATP5B. ATP5B is an ATP synthase in the mPTP complex, which could regulate mPTP opening by binding CypD.^2^ Therefore, we found that GCN5L1 induced mROS release and increased the cytoplasmic ROS. These results demonstrated that GCN5L1 could regulate the mitochondrial signalling transmitting to the cytoplasm.

Emerging evidence suggested that abnormal metabolic signals and iron homeostasis are the main causes of ferroptosis, which is vital in regulating the progression of liver disease.^55^ Ferroptosis induced the release of DAMPs that established an inflammatory microenvironment including neutrophils and NETs. In the past decade, NETs have been proven to play an important role in non‐infectious inflammation.^56^ Several works have demonstrated that NETs promote inflammation, which might recruit multiple immune cells.^45^, ^57^ ^–‐^ ^59^ Inflammatory cells could trigger several signals including inflammatory molecules, lipid messengers, ROS and endoplasmic reticulum stress, which perturbs hepatocytes and causes metabolic abnormality, which promotes NAFLD progression.^47^, ^60^, ^61^ Works also demonstrate that NETs could promote HCC progression by inducing inflammatory microenvironments.^45^, ^62^ In this study, we found that GCN5L1 triggered ferroptosis and released HMGB1, which increased the infiltration of neutrophils and promoted the formation of NETs. The deletion of NETs improved the GCN5L1‐induced NASH progression. This work found the novel roles of GCN5L1 in regulating NASH progression and could be a target in the treatment of NASH. A study has demonstrated that GCN5L1 deletion in hepatocytes exhibits increased lysosomal lipid uptake and lipolysis,^63^ which might diminish hepatocyte lipid stores and provide another explanation for GCN5L1‐induced NAFLD progression.

In this study, we mainly focused on the function of GCN5L1 in hepatocytes. However, whether the GCN5L1 could regulate the NASH progression in nonparenchymal cell‐like macrophages and hepatic stellate cells and the underlying mechanisms need further study. Furthermore, exploring the drugs that target GCN5L1 is promising, and could provide a potential treatment option for NASH. In summary, we showed that GCN5L1 could regulate oxidative metabolism and establish an inflammatory microenvironment in promoting NASH progression, which provides a potential therapeutic target for immunometabolic diseases.

## CONFLICT OF INTEREST STATEMENT

The authors declare no conflict of interest.

## FUNDING INFORMATION

This work was supported by the Clinical Science and Technology Innovation Program of Jinan (202134039), the Natural Science Foundation of Shandong Province (ZR2022QH070 and ZR2022QH349) and the National Natural Science Foundation of China (82203654).

## Supporting information



Supporting Information

Supporting Information

Supporting Information

Supporting Information

Supporting Information

Supporting InformationS1
