# Supplementary figures and images for: Mitochondrial general control of amino acid synthesis 5 like 1 promotes nonalcoholic steatohepatitis development through ferroptosis‐induced formation of neutrophil extracellular traps
Source: Clin Transl Med. 2023 Jul 6;13(7):e1325. doi: 10.1002/ctm2.1325 (PMC10326373; doi:10.1002/ctm2.1325)

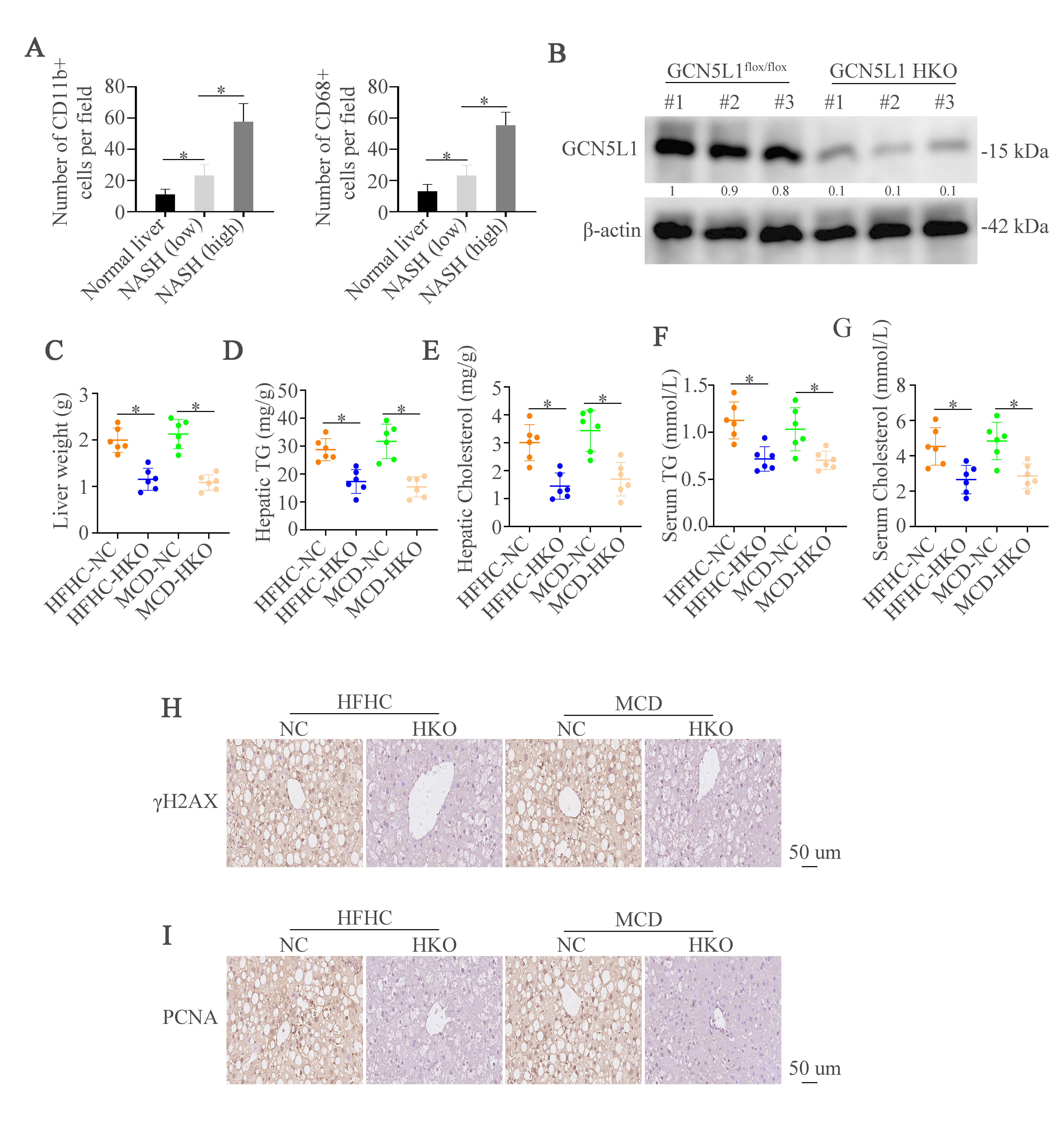

Supplement: Supplementary file 1 — Supporting Information [file CTM2-13-e1325-s001.tif]

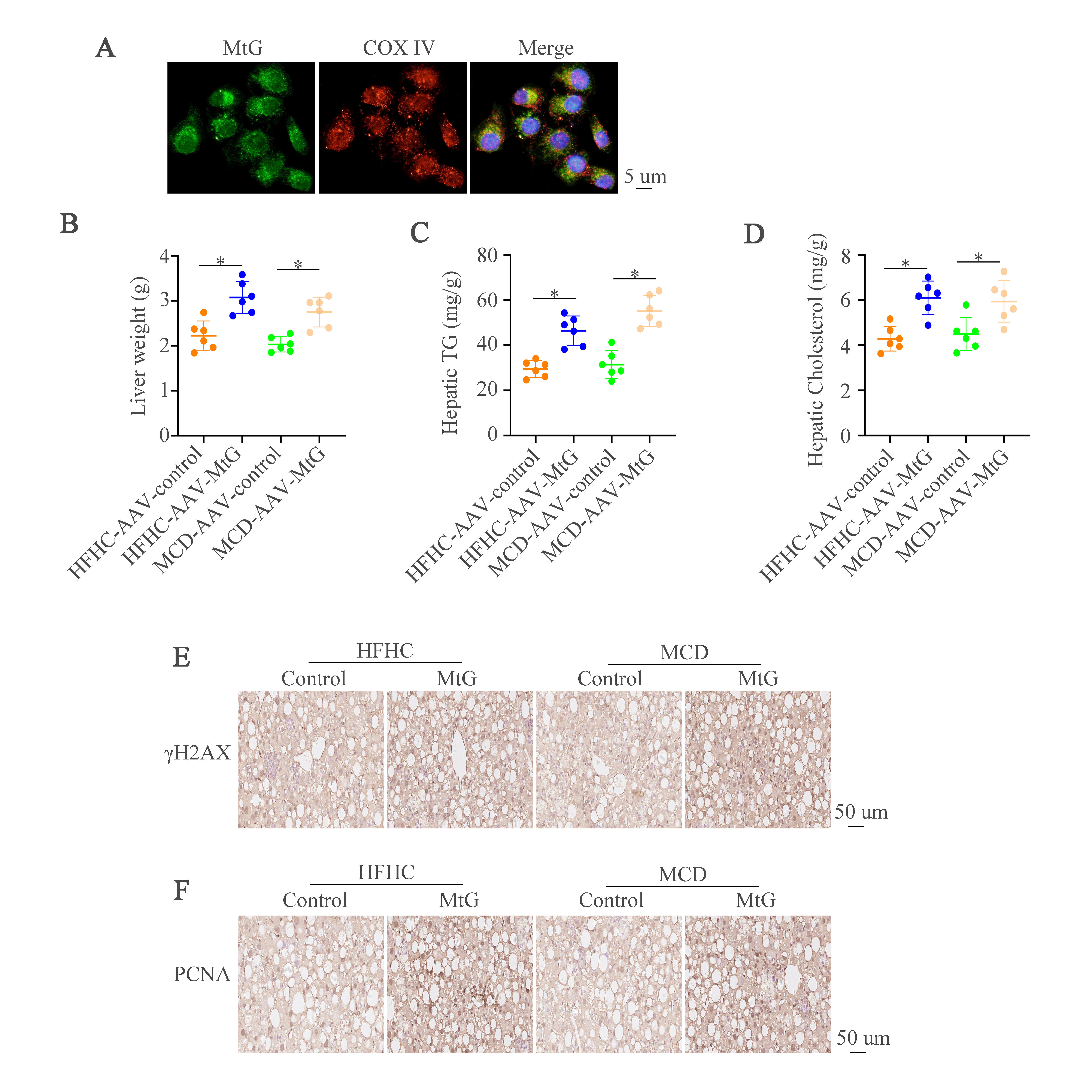

Supplement: Supplementary file 2 — Supporting Information [file CTM2-13-e1325-s004.tif]

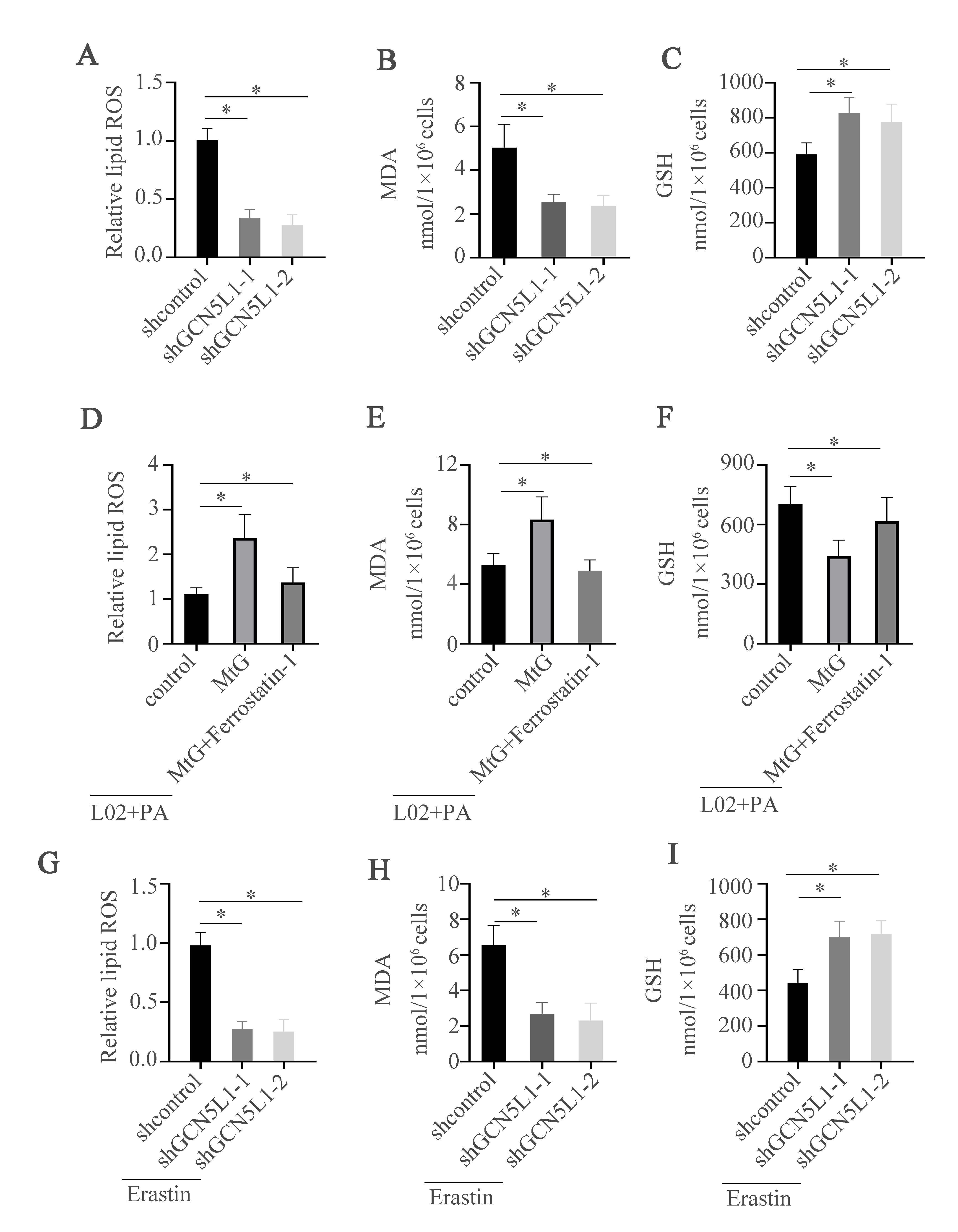

Supplement: Supplementary file 3 — Supporting Information [file CTM2-13-e1325-s003.tif]

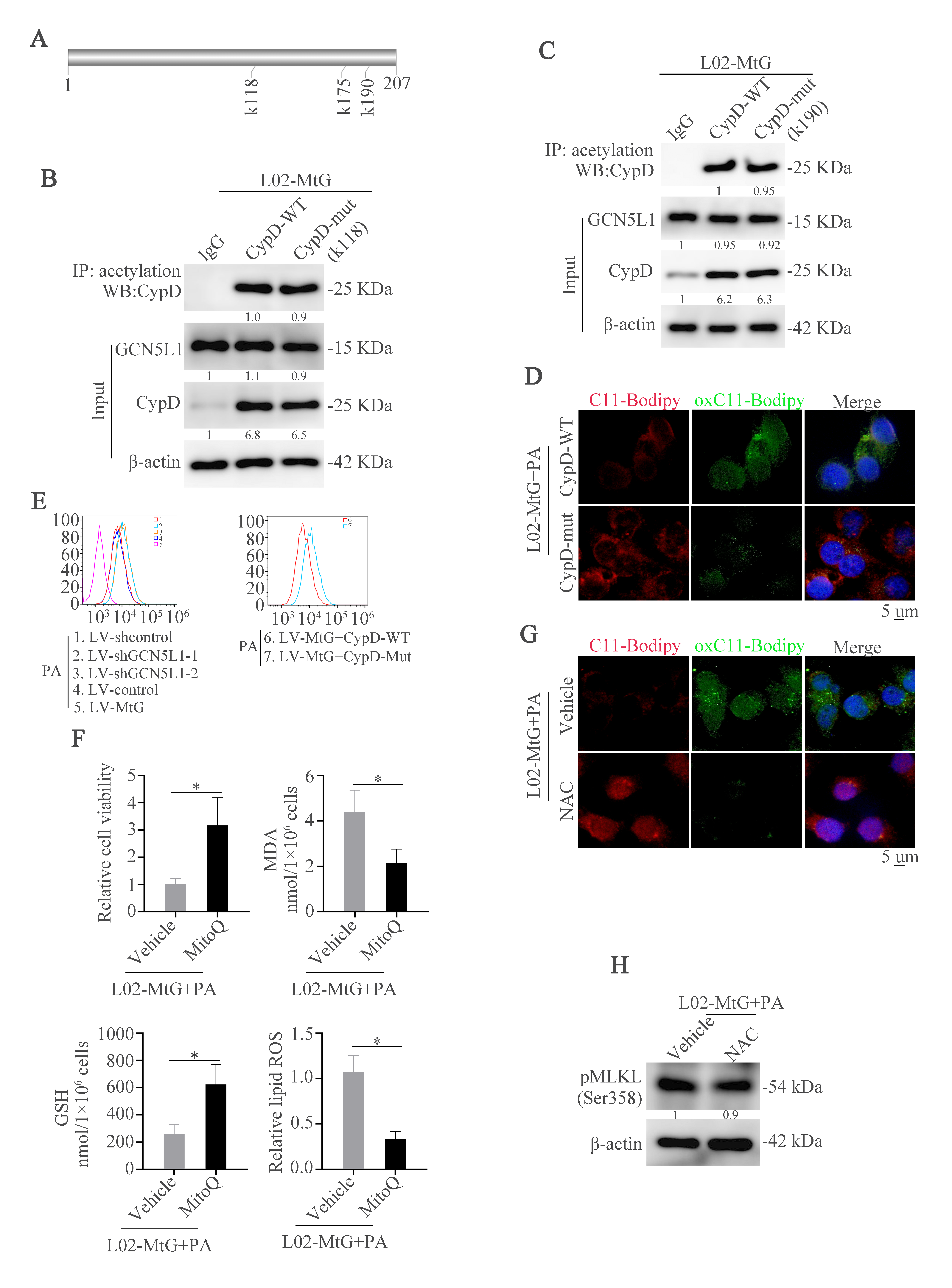

Supplement: Supplementary file 4 — Supporting Information [file CTM2-13-e1325-s002.tif]

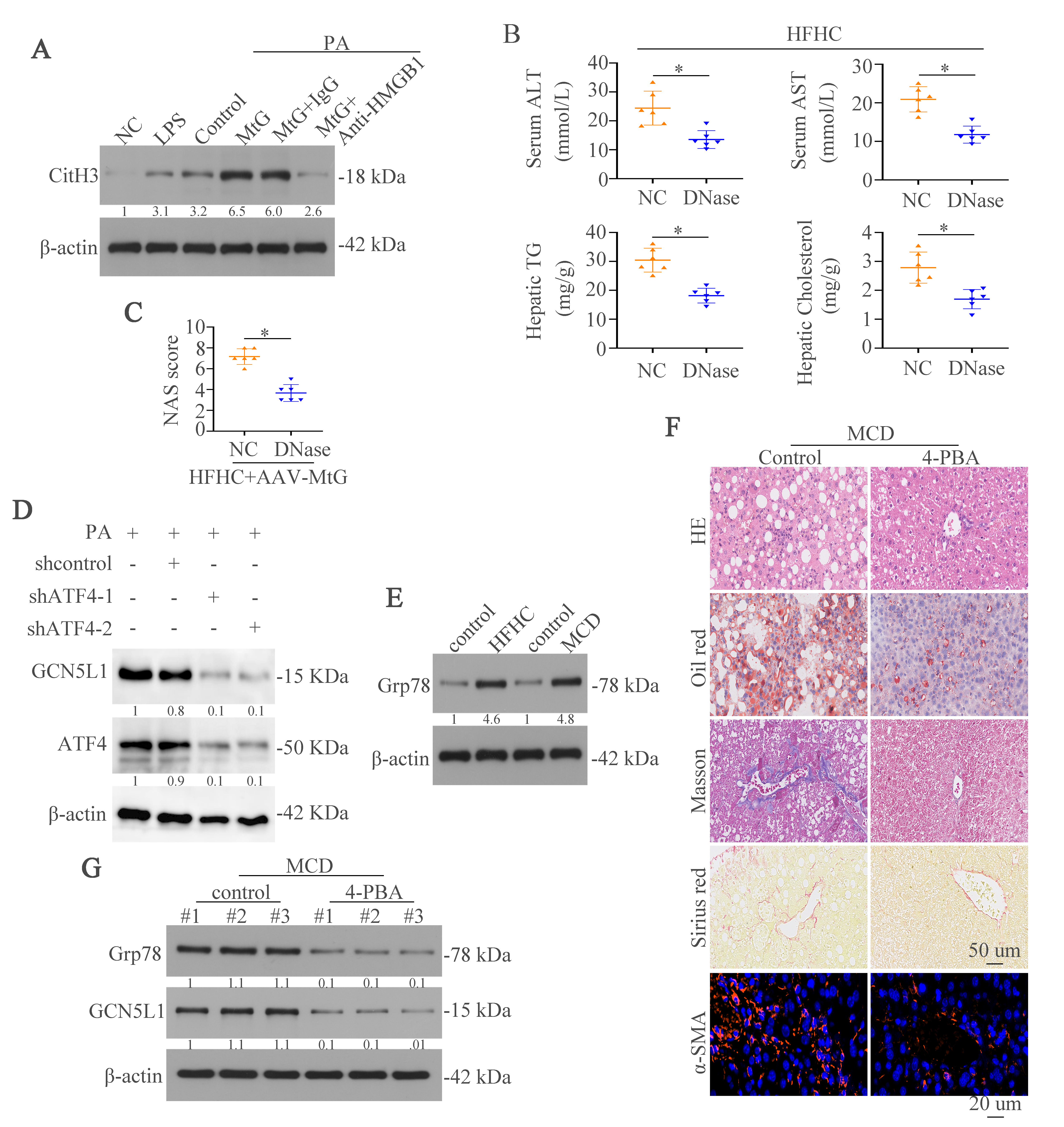

Supplement: Supplementary file 5 — Supporting Information [file CTM2-13-e1325-s006.tif]
