# Supplementary material for: Mitochondrial general control of amino acid synthesis 5 like 1 promotes nonalcoholic steatohepatitis development through ferroptosis‐induced formation of neutrophil extracellular traps
Source: Clin Transl Med. 2023 Jul 6;13(7):e1325. doi: 10.1002/ctm2.1325 (PMC10326373; doi:10.1002/ctm2.1325)
Supplement: Supplementary file 6 — Supporting InformationS1 [file CTM2-13-e1325-s005.docx]

**Supplementary material**

**Methods**

**Cell culture**

Immortalized liver cell line (HL-7702) was purchased from the Institute of Biochemistry and Cell Biology, Chinese Academy of Science, China. Hepa1-6 cells were purchased from the Institute of Biochemistry and Cell Biology, Chinese Academy of Science, China. Cells were cultured in Dulbecco’s Modified Eagle Medium (DMEM) 37°C in a 5% CO2 incubator. The medium was supplemented with 10% FBS, 100μg/ml penicillin, and 100μg/ml streptomycin. These above cell lines were authenticated by short tandem repeats (STRs) DNA profiling. All cells were tested for mycoplasma contamination before use with the Universal Mycoplasma Detection Kit (ATCC 30-1012K) and were not contaminated by mycoplasma.

**Primary hepatocytes isolation**

Mouse primary hepatocytes were isolated from 8-week-old C57BL/6J male mice as described below^1^. Briefly, mice were anesthetized with 3% pentobarbital sodium and then were perfused through the portal vein with Liver Perfusion Medium followed by Liver Digestion Medium. After digestion, the liver was excised, minced, filtered through a 100 mm steel mesh. Hepatocytes were separated after two centrifugations at 50g for 1 min. The isolated hepatocytes were cultured in DMEM supplemented with 10% FBS and 1% penicillin/streptomycin for other tests.

**Detection of NETs in *vitro* and in *vivo***

Anti-citrullinated histone-3 (CitH3, 1:1000, Abcam) was used to detected the NETs in tissues and cells samples by western blot or immunofluorescence (IF). To quantify NETs in mouse serum and cell culture supernatant, a capture ELISA to detect myeloperoxidase (MPO) associated with DNA (MPO-DNA) was performed as described previously ^2^.

**Cell treatment**

The cells in this study were stimulated with the elements as following: Erastin (2 μM), palmitic acid (0.5mmol/L), ferrostatin-1 (2 μm) and LPS at 100 nm.

**Immunohistochemistry**

IHC was performed on 4-μm-thick, routinely processed paraffin-embedded sections. Briefly, the tissue sections were deparaffinized after baking at 60 °C for an hour. Endogenous peroxidase activity was blocked by 3% (vol/vol) hydrogen peroxide in methanol for 12 min and washes with PBS. Then the slides were immersed in 0.01 mol/L citrate buffer solution or EDTA for 30 min using a microwave. Primary antibody was added in the tissues in 4 °C for overnight. The tissue microarrays were stained for GCN5L1 (Proteintech, 19687-1-AP), PCNA (cell signaling technology, # 13110), and γH2AX (cell signaling technology, # 9718). The sections were treated with a peroxidase-conjugated second antibody (Santa Cruz) for 30 min at room temperature and then washed with PBS. Reaction product was visualized with diaminobenzidine for 2 min. Images were obtained under a light microscope (Olympus, Japan) equipped with a DP70 digital camera. Analyses were performed by two independent observers who were blinded to the clinical outcome. The immunostaining intensity score was briefly described as following. Analyses were performed by two independent observers who were blinded to the clinical outcome.

**Immunofluorescence (IF)**

Formalin-fixed paraffin-embedded sections (4μm) were baked, deparaffinized, rehydrated, followed by antigen retrieval and permeabilized. After that the tissues were blocked with 10% goat or donkey serum for 30 minutes and incubated with primary antibodies at 4°C overnight. After washing, appropriate secondary antibodies were used. The DAPI was used to stain cell nucleus for ten minutes. Florescence was visualized under an Olympus fluorescence microscope.

Antibodies used in Immunofluorescence

| Antibodies | Source |
| --- | --- |
| Anti-αSMA | Abcam, ab124964 |
| Anti-CD11b | Cell signaling technology, # 17800 |
| Anti-CD68 | Cell signaling technology, # 26042 |
| Anti-F4/80 | Cell signaling technology, #30325 |
| Anti-Calcein | Abcam, ab141420 |
| Anti-CitH3 | Abcam, ab281584 |
| Anti-MPO | Cell signaling technology, #14569 |
| Anti-Ly6G | Invitrogen, 14-5931-81 |

**Quantitative Real-time PCR (RT-qPCR)**

The RNeasy Plus Mini Kit (50) kit (Qiagen, Hilden, Germany) was used to extract total RNA, which was then reverse transcribed with the Advantage RT-for-PCR Kit (Qiagen) in accordance with the manufacturer’s protocols. The target sequence was amplified with RT-qPCR with the SYBR Green PCR Kit (Qiagen). The cycling parameters used were 95 °C for 15 s, 55-60 °C for 15 s, and 72 °C for 15 s for 45 cycles. The 2^–ΔΔCt^ method was used to determine relative fold changes in target gene expression. All experiments were performed in triplicate.

**Mitochondria isolation**

The mitochondrial fraction was prepared using a commercial kit (cat# SM0020, Solarbio, China) in accordance with the manufacturer’s protocol.

**Co-immunoprecipitation (Co-IP)**

For Co-IP, tissues or cells were lysed in Pierce IP lysis buffer (No. 87787, Thermo Fisher) and protease inhibitor cocktail (No. 04693159001, Roche). Lysates (500 μl) were incubated with anti-GCN5L1 antibody (Proteintech 19687-1-AP) or control IgG (40 μl Protein A/G PLUS-Agarose, No. 17061801, GE health) overnight at 4 °C. The beads were washed three times with PBS, followed by western blot.

**Immunoprecipitation Assay**

Immunoprecipitation (IP) assay was carried out using a Crosslink IP kit (26147, Thermo Scientific Pierce) according to the manufacturer’s instructions. In short, after treated with proteasome inhibitor MG132 (#2194, CST,10 nM) for 6∼8 h, cells were lysed with IP Lysis Buffer. Lysates (1 mg) were incubated at 4◦C overnight with 3 mg (rabbit anti-acetylation antibody or rabbit control IgG antibody) and 20 ml Protein A/G Agarose on a rotator. The eluent was analyzed by western blot.

**Western blot analysis**

Proteins from lysed cells were fractionated by SDS-PAGE and transferred to nitrocellulose membranes. Nonspecific binding sites were blocked with 5% BSA in TBST for 2 hours at room temperature. Blots were incubated with a specific antibody overnight at 4 °C. The membranes were then washed with TBST 3 times and incubated with an HRP-conjugated secondary antibody. Proteins were visualized using an ImmobilonTM Western Chemiluminescent HRP substrate (Millipore, USA).

The primary antibodies used are listed below.

| Antibodies | Source |
| --- | --- |
| anti-GCN5L1 | Proteintech, 19687-1-AP |
| anti-β-actin | Proteintech, 60008-1-Ig |
| anti- COX IV | Cell signaling technology, # 4850 |
| anti-p-ERK1/2(T202/Y204) | Cell Signaling Technology, #4370 |
| anti-ERK | Cell Signaling Technology, #4695 |
| anti-JNK | Abcam, ab179461 |
| anti-JNK (phosphoT183+T183+T221)) | Abcam, ab124956 |
| anti-p38 | Cell Signaling Technology, #8690 |
| anti-p-p38(Thr180/Tyr182) | Cell Signaling Technology, #4511 |
| anti-CypD | Cell signaling technology, #43603 |
| anti-GRP78 | Cell signaling technology, # 3177 |
| anti-PERK | Cell signaling technology, # 5683 |
| anti-pPERK | Cell signaling technology, # 3179 |
| anti-pEIF2α | Cell signaling technology, #3396 |
| anti-ATP5B | Abcam, ab14730 |
| anti-CitH3 | Abcam, ab5103 |
| anti-ATF4 | Abcam, ab184909 |

**RNA-seq and data processing**

The RNA-seq was analyzed by Oebiotech (Shanghai, China). The process was described below. After total RNA isolation and quality verification, 200 ng of RNA from each sample was used for cDNA library construction using the MGIEasy RNA Library Prep Kit following the manufacturer’s protocol. HISAT2 software (version 2.21) was used to align clean reads based on Ensembl reference genomes. SAMtools (version 1.4) was used to sort and convert the mapped reads to BAM format. StringTie (version 1.3.3b) was used to identify the fragments per kilobase per million (FPKM) and read counts. Finally, DESeq2 was used to calculate differential gene expression and statistical significance.

**Co-immunoprecipitation and mass spectrometry**

Protein extraction and purification were performed using Pierce™ Co-Immunoprecipitation Kit (26149, Thermo Fisher Scientific). Mass spectrometry was performed by Oebiotech (Shanghai, China).

**Cell viability analysis**

Cell viability was evaluated with an alamarBlue Cell Viability Assay Kit (MedChemExpress, Monmouth Junction, NJ, USA) according the manufacturer’s instructions. In brief, cells were plated in a 96-well plate and exposed to various stimulus for indicated times. The alamarBlue Reagent (10 μl) was added to each well and incubated at 37°C in 5% CO2 for one hours, and then the plates were measured at 450 nm. Average percentage of inhibition at each concentration was calculated.

**Lipid peroxidation assay**

The relative malondialdehyde (MDA) concentration in cell lysates was assessed using a Lipid Peroxidation (MDA) Assay Kit (#ab118970, Abcam) according the manufacturer’s instructions.

**Glutathione assay**

The relative glutathione (GSH) concentration in cell lysates was assessed using a Glutathione Assay Kit (#CS0260, Sigma) according the manufacturer’s instructions.

**GPX activity**

GPX activity was measured using their respective assay kits (Beyotime Institute of Biotechnology). Briefly, cells were cultured in 10 cm^2^ plates overnight and treated with different stimulus. After treatments, cells were washed with PBS and collected to measure intracellular GPX activity in accordance with the manufacturer's instructions.

**ELISA**

Concentrations of HMGB1 in the culture supernatant of cells were determined using a Human ELISA Kit (#ARG81185, Arigo) according to the manufacturer’s instructions.

**Neutrophil migration**

Human neutrophils were isolated from peripheral blood obtained from healthy controls according to the manufacturer's instructions (P9040; Solarbio; Tongzhou, Beijing, China) and maintained in DMEM with 10% FBS for further use. For migration analysis, the neutrophils (1 × 10^5^) were seeded in the upper chamber and 600 ul conditioned medium from the indicated cells was placed in the lower chamber of Transwell systems (Corning) for 24h. The number of neutrophils was counted under the microscope.

**Plasmid construction**

Plasmid construction was performed according to standard procedures. For example, (-1875/+45) *GCN5L1*, was generated from human genomic DNA. This construct corresponds to the sequence from -1875 to +41 (relative to the transcriptional start site) of the 5’-flanking regions of human *GCN5L1* gene. It was generated with forward and reverse primers at the 5’ and 3’-ends, respectively. The polymerase chain reaction (PCR) product was cloned into the pGL3-Basic vector (Promega). The ATF4 binding sites in the *GCN5L1*promoter were mutated using the QuikChange II Site-Directed Mutagenesis Kit (Stratagene). The constructs were confirmed by DNA sequencing. Other promoter constructs were cloned in the same manner.

**Construction of lentivirus and stable cell lines**

Lentiviral vectors encoding shRNAs were generated using PLKO.1-TRC (Addgene) and designated as LV-shATF4, LV-shGCN5L1 and LV-shcontrol. “LV-shcontrol” is a non-target shRNA control. The vector “pLKO.1-puro non-Target shRNA Control Plasmid DNA” (purchased from Sigma, SHC016) contains an shRNA insert that does not target any known genes from any species. Mitochondria-GCN5L1 (MtG) was generated by fusing the GCN5L1 encoding sequence with the COX 8 mitochondrial targeting sequence (36 amino acid at N terminal) and 3×-flag sequence ^3^. Lentiviral vectors were constructed in FUW-teto (Addgene) and designated as LV-MtG. An empty vector was used as the negative control and was designated as LV-control. Concentrated lentivirus was transfected into the cells with a multiplicity of infection (MOI) ranging from 30 to 50 in the presence of polybrene (6 μg/ml). Seventy-two hours after infection, cells were selected for 2 weeks using 2.5 μg/ml puromycin (OriGene). Selected pools of knockdown and over-expressing cells were used for the following experiments.

**Transient transfection**

The cells were plated at a density of 1×10^5^ cells/well in a 24-well plate. After 12-24 hours, the cells were co-transfected with 0.6 μg of the expression vector plasmids, 0.18 μg of the promoter reporter plasmids, and 0.02 μg of the pRL-TK plasmids using Lipofectamine 2000 (Invitrogen, USA) according to the manufacturer’s instructions. After 5 h of transfection, the cells were washed and allowed to recover overnight in fresh medium supplemented with 1% FBS for 48 h. Serum-starved cells were used for the assay.

**Luciferase reporter assay**

Luciferase activity was detected using the Dual Luciferase Assay (Promega, USA) according to the manufacturer’s instructions. The transfected cells were lysed in culture dishes containing a lysis buffer and the resulting lysates were centrifuged at maximum speed for 1 min in a microcentrifuge. Relative luciferase activity was determined using a ModulusTM TD20/20 Luminometer (Turner Biosystems, USA), and the transfection efficiencies were normalized according to the Renilla activity.

**Chromatin immunoprecipitation Assay (ChIP)**

1% formaldehyde was used to crosslink the treated cells at 37 °C for 10 min and then the cells were resuspended in 300 μl of lysis buffer. The DNA was sheared to small fragments by sonication. Sonicated chromatin was diluted to a final SDS concentration of 0.1% and aliquots were rotated with antibody O/N at 4 °C. The recovered supernatants were incubated with specific antibodies or an isotype control IgG for 2 h in the presence of herring sperm DNA and Protein A/G Magnetic beads (Thermo Fisher). The antibody is anti-ATF4 (Abcam, ab85049). The immunoprecipitated DNA was retrieved from the beads with 1% SDS and a 1.1 M NaHCO3 solution at 65 °C for 6 hr. The DNA was then purified using a PCR Purification Kit (QIAGEN, USA).

**Statistical analysis**

All values were recorded as the mean ± standard deviation (s.d.). All experiments were repeated three or more independent biological replicates. Statistical significance between the means of two groups was determined using Student’s t tests (normal distribution), Mann–Whitney U tests (abnormal distribution) or Wilcoxon signed rank test (matched pairs). The statistics of the means of multiple groups were performed using one way ANOVA or two-way ANOVA. Immunohistochemical score was analyzed by chi-squared test. The cumulative recurrence and survival curves were shown by the Kaplan–Meier method and the statistical significance were determined by log-rank test. Multivariate analysis was performed by Cox regression analysis. Correlations were performed by using a Pearson correlation test. Statistical analysis was justified as appropriate among all figures. P values < 0.05 were considered to be statistically significant. Statistical values were calculated with SPSS software (Version 20.0) or GraphPad Prism 8.0 software.

**References**

1 Yang X, Sun D, Xiang H, Wang S, Huang Y, Li L *et al*. Hepatocyte SH3RF2 Deficiency Is a Key Aggravator for NAFLD. Hepatology 2021; 74: 1319-1338.

2 van der Windt DJ, Sud V, Zhang H, Varley PR, Goswami J, Yazdani HO *et al*. Neutrophil extracellular traps promote inflammation and development of hepatocellular carcinoma in nonalcoholic steatohepatitis. Hepatology 2018; 68: 1347-1360.

3 Wang L, Scott I, Zhu L, Wu K, Han K, Chen Y *et al*. GCN5L1 modulates cross-talk between mitochondria and cell signaling to regulate FoxO1 stability and gluconeogenesis. Nat Commun 2017; 8: 523.

**Supporting figure legends**

**Supporting Figure 1.**

(A) Quantitative analysis of number of CD11b^+^ and CD68^+^ cells in figure 1E.

(B) Western blot analyzed the expression of GCN5L1 and β-actin in indicated mice.

(C-E) Graphs showed the liver weight, hepatic TG and cholesterol in mice (n= 6/group).

(F-G) Graphs showed the serum TG and cholesterol in mice (n= 6/group).

(H-I) IHC staining of γ-H2AX and PCNA was shown.

**Supporting Figure 2.**

(A) IF was used to detect the location of GCN5L1. Blue: MtG, Red: COX IV.

(B-D) Graphs showed the liver weight, hepatic TG and cholesterol in mice (n= 6/group).

(E-F) IHC staining of γ-H2AX and PCNA was shown.

**Supporting Figure 3.**

(A-C) Lipid ROS, MDA and GSH were assayed in L02 cells transfected with LV-shcontrol or LV-shGCN5L1.

(D-F) Lipid ROS, MDA and GSH were assayed in indicated cells.

(G-I) Lipid ROS, MDA and GSH were assayed in L02 cells transfected with LV-shcontrol or LV-shGCN5L1 and treated with erastin.

**Supporting Figure 4.**

(A) The acetylation sites in CypD.

(B) Co-IP was used to show the acetylation of CypD in L02-MtG cells transfected with WT or mutation plasmid in k118 of CypD.

(C) Co-IP was used to show the acetylation of CypD in L02-MtG cells transfected with WT or mutation plasmid in k190 of CypD.

(D) Lipid ROS levels were detected by C11-BODIPY fluorescence in L02-MtG cells treated with CypD WT or CypD mut under the PA treatment.

(E) Flow cytometry showed the mPTP opening by calcein loading in L02 cells transfected with LV-shcontrol, LV-shGCN5L1, LV-control, LV-MtG, CypD-WT or CypD-Mut under PA treatment.

(F) Relative cell viability, MDA, GSH and Lipid ROS were assayed in L02-MtG cells with or without MitoQ under PA treatment.

(G) Lipid ROS levels were detected by C11-BODIPY fluorescence in L02-MtG cells treated with vehicle or NAC under the PA treatment.

(H) Western blot analyzed the expression of pMLKL and β-actin in indicated cell.

**Supporting Figure 5.**

(A) Western blot showed the CitH3 expression in different groups.

(B) Graphs showed the serum ALT, AST, hepatic TG and cholesterol in mice (n= 6/group).

(C) NAS score was shown in different groups.

(D) Western blot analyzed the expression of GCN5L1, ATF4 and β-actin in indicated HCC cells.

(E) Grp78 level was showed in control and NASH models.

(F) H&E, Oil Red O, Masson, Sirius Red, and α-SMA staining were shown in WT or GCN5L1 HKO mice fed with HFHC, or MCD diet.

(G) Grp78 and GCN5L1 expression in mice treated with control or 4-PBA.
